# A “Mean Quarrelsome Spirit:” Controversy in British Systematics, 1822–1836

**DOI:** 10.1007/s10739-023-09743-7

**Published:** 2023-12-20

**Authors:** Jordan Thomas Mursinna

**Affiliations:** Department of History, University of California, Berkeley, 3229 Dwinelle Hall, Berkeley, CA 94720-2550 USA

**Keywords:** Systematics, Scientific controversy, Quinarian system, William Sharp Macleay, Charles Darwin

## Abstract

British systematics was distinctly marked by a raft of vituperative controversies around the turn of the 1830s. After the local collapse of broad consensus in the Linnaean system by 1820, the emergence of new schemes of classification—most notably, the “quinarian” system of William Sharp Macleay—brought with it an unprecedented register of public debate among zoologists in Britain, one which a young Charles Darwin would bitterly describe to his friend John Stevens Henslow in October 1836 as possessing a “mean quarrelsome spirit,” conducted in “a manner anything but like that of gentlemen.” This article aims to provide a social and conceptual account of the remarkable tenor of zoological discourse in Britain in the late 1820s and early 1830s, with joint attention to the philosophical and interpersonal commitments at play. In doing so, it analyzes the three of the period’s most striking public controversies, each of which counted key advocates of the quinarian system as central participants.

## Introduction

In 1822, one year after the publication of the second part of his *Horae entomologicae*—which laid out the elements of his “quinarian” system of classification in exhaustive detail—the Scottish entomologist William Sharp Macleay turned his attention to the dissemination of his ideas. The Linnean Society of London, to which he had been recently inducted, did not prove a receptive audience. In the early decades of the 19th century, the Society was dominated not only by botanists, but by what Gordon McOuat has described as a contingent of “conservative” naturalists who staunchly opposed any substantial reform of the taxonomic groups laid out by their organization’s namesake (Desmond 1985a; McOuat 1996, p. 478). The quinarian system—which radically rearranged the purported order of nature into a harmonious series of interconnected circles, each containing precisely five members—was precisely the sort of work the conservative Linneans wished to discredit. In response, Macleay took a leading role in founding the Zoological Club of the Linnean Society, established in 1823 and designed to be a home for the growing numbers of “radical anti-Linnaeans” seeking to push systematic natural history in new directions (McOuat 1996, p. 478).

As the popularity of the quinarian system grew, divisions formed within the Club between its advocates and those of a rival classification scheme—the “dichotomous” system of botanist Adrian Hardy Haworth. At the same time, another contingent was forming; sensing the imminent collapse of a broad Linnaean consensus as new systems like the quinary and dichotomous proliferated, a new generation of conservatives lead by James Ebenezer Bicheno renewed calls for a preservation of Linnaean principles and moderation in taxonomic reform. Towards the end of the decade, attendance at meetings of the Zoological Club, sharply divided along ideological lines and bereft of finances, waned. Though the Club officially dissolved in 1830, just seven years after its official formation, a familiarity with its internal factionalization is crucial in understanding the controversy and chaos that would so distinctly characterize British zoology around 1830.

The role of Macleay and his immensely-popular quinarian system in pre-Darwinian natural history has constituted a growing area of interest for scholars in recent decades. Rose Novick has shown that Macleay’s system, far from the quasi-numerological oddity that it has been portrayed as (De Beer 1963, p. 13; Ghiselin 1969, p. 104; Blaisdell 1982, p. 24), in fact provided a sophisticated response to key conceptual issues in the philosophy of classification at the time (Novick 2016, pp. 110-117). She, along with Dov Ospovat and Mario Di Gregorio, has also shown that Charles Darwin took the system seriously, and its central tenets served as important reference points in his early thinking about evolution (Ospovat 1981, pp. 101–113; Di Gregorio 1996; Novick 2019). And while Gordon McOuat has demonstrated the system’s centrality to the short life of romantic thought in early nineteenth century Britain (McOuat 2017, pp. 171–176), far more consequential was its role in prompting the nomenclatural reforms of Hugh Edwin Strickland in the early 1840s (McOuat 1996). Even long after its subsequent fall from popularity in Britain, Jennifer Coggon has revealed traces of its influence in late-19th century North America (Coggon 2002).

Less has been written, however, on the period between the quinarian system’s genesis in 1819 and its fall from grace at the hands of Strickland and his allies in the 1840s; to date, Adrian Desmond’s great institutional histories of the Zoological Club of the Linnean Society and the Zoological Society of London remain the best resource (Desmond 1985a, b). More recently, David Lowther has re-opened on the subject, with a focus on controversy and print culture (Lowther 2020). Questions remain: how is one to account for the eruption of such fierce controversies in British systematics between 1829 and 1831, and why were quinarians so frequently involved? In suggesting certain answers, this article first introduces Macleay’s two closest allies in promoting the quinarian system, and the two major theoretical factions that emerged within the Zoological Club to rival it. It then recounts and analyzes three acerbic and widely-publicized controversies that Macleay, his allies, and members of those rival factions became entangled in—to both the distaste and fascination of Britain’s natural historical readership.

## The Early Quinarians

It is well documented throughout the natural historical literature of the 1820s that the quinarian system rapidly generated significant interest in England. And though Macleay’s work received a number of favorable appraisals, even skeptics often recognized it as an original, rigorous, and provocative work of natural history developed—for the first time in recent memory—by an Englishman (e.g. Vigors 1825; Lindley 1826).^1^ But among its many reviewers, two working naturalists in London were most rapidly convinced of its truth: the ornithologists Nicholas Aylward Vigors and William Swainson. Both men would take up the quinary system in the mid-1820s, and both would remain convinced of its essential truth throughout their scientific careers. Any study of quinarian systematics in England, therefore, cannot merely consider the writing of William Sharp Macleay in isolation; both Vigors and Swainson played intimate roles in its institutionalization, dissemination, explication, and ultimately, in its decline.

Vigors, an Irishman from County Carlow, is the first known naturalist to embrace Macleay’s quinary system. Born in 1785 or 1787 (records differ) Vigors entered Trinity College, Oxford in 1803, where he remained without completing his degree until he joined the Grenadier Guards to fight the *Grande Armée* in Iberia (Long 2009). When he took a wound at the Battle of Barossa in early 1811, Vigors subsequently returned to Oxford, where he finished his BA in 1817 and MA in 1818, after spending no fewer than 13 years at the university. Following his graduation, Vigors moved to London, and was inducted a Fellow of the Linnean Society in 1819. It is not clear at what point Vigors took an interest in ornithology, but he published on little else throughout his life, and must have possessed some significant knowledge of the subject by the time of his induction. His long years spent at Oxford would have no doubt provided him ample time to familiarize himself with it.

There is little written correspondence between Vigors and Macleay; either the letters have been lost, or the two communicated verbally at and after meetings of the Linnean Society. Regardless, Vigors had evidently adopted the quinary system by the end of 1823, when he presented a paper to the Zoological Club on December 9th on the “Natural Affinities that connect the Orders and Families of Birds” that ubiquitously followed a quinarian arrangement. In the introduction to the paper (which stood at over 122 pages) Vigors heaped praise on Macleay, conceding that although his countrymen had recently been “chiefly indebted” to the French for progress in zoology,

Great Britain, however, has made ample amends for her tardy adoption of the more philosophical views of the science…. It has been reserved for one of her sons to throw a new light upon the sphere of animated Nature, and to bring to view a principle that pervades her works, as beautiful as it is comprehensive. In the year 1819, the enlightened author of the *Horae Entomologicae* first called attention of the lovers of science to a principle which he discovered in a minute group of insects, and which, with a comprehensiveness of mind, and an accuracy of execution, seldom united in an individual, he subsequently followed up through the whole range of animal life. (Vigors 1825, p. 397)

Vigors’s stated admiration for Macleay went beyond nationalistic pride; he proclaimed that Macleay’s theory had effected a “great revolution” in zoology that “raised the science to that elevated rank among the subjects of human research.” Because of Macleay, “[t]he investigation of Nature has ceased to be a mere work of observation” but now revealed “grand and sublime combinations…in the great system of Nature” (Vigors 1825, p. 397). His celebration did not stop there; Vigors further compared Macleay to the great natural philosophers of the 17th century, remarking that the quinary theory had “wrought the same change as may be supposed to have affected the views of the early astronomer, when his attention was withdrawn from the mere observation of the splendid orbs of the firmament… to the more sublime contemplation of the harmonious system in which they revolve through infinite space” (Vigors 1825, p. 398). While Vigors did not mention Newton by name (which would perhaps have been too high praise, even given his warm sentiments) the implicit comparison is apparent. Given his nationalistic framing of Macleay’s impact (and his injury at French hands a dozen years prior) there are reasons to suggest that the narrative of a national return to form in the sciences bore significant appeal to Vigors.

Vigors’s expertise in ornithology was an asset to the broader quinarian project—while Macleay had designated *Aves* as a class of the division *Vertebrata* in the second part of the *Horae entomologicae*, he had not published on their particular subdivision into orders, and his newfound ally outstripped his knowledge of the class significantly. Indeed, in his working notes on birds, Macleay often refers to Vigors’s opinions, typically seeking to corroborate them with quantitative data like the number of vertebrae or the lengths of varying sections of the digestive tract.^2^ And though it is uncertain who produced their primary arrangement first, Macleay’s five orders of birds in his drafts—birds of prey, perching birds, wading birds, ground-feeding birds, and web-footed birds—mirror Vigors’s exactly.^3^ Moreover, Vigors was not just Macleay’s bird-expert and loudest supporter; he became deeply involved in the organizational and political wrangling that Macleay undertook immediately after the publication of part two of the *Horae entomologicae*. Letters from William Kirby to Macleay in 1822 reference Vigors’s regular encouragement for Kirby to support the formation of a zoological organization within the Linnean Society.^4^ And once such an organization had been formed, Vigors would take the roles of both its secretary and chairman before its ultimate dissolution in 1830.

William Swainson the son of a customs clerk from Liverpool, was slower to adopt the quinary theory—but once he had, he did so with no less enthusiasm than Vigors. Born in 1789, Swainson was three years Macleay’s senior, though his family was considerably Macleay’s junior in wealth and esteem. Swainson lacked the familial support or social connections to pursue natural history freely or secure prestigious positions in law or government, as Vigors and Macleay respectively enjoyed (Knight 1985; Natusch 2004). Rather, he would prove to be one of England’s earliest career naturalists, supporting himself and his family almost entirely from his zoological publications. Yet Swainson was not entirely bereft of social rank, as demonstrated by his prestigious affiliations and friendships. In 1816, at the age of 26, he was elected a member of the Linnean Society. Four years later, at the recommendation of England’s most famous living naturalist, Sir Joseph Banks, he became a Fellow of the Royal Society. How did Swainson, born in Liverpool, lacking an Oxbridge education—or any college education for that matter—achieve this? Part of the answer can be found in letters sent from Swainson to powerful naturalists throughout the 1810s.

Swainson, in short, had a model for ingratiating himself with powerful figures in natural history. As Beth Fowkes Tobin relates, his father John Timothy Swainson had impressed Margaret Cavendish Bentinck, the Duchess of Portland and an avid collector of shells, with an impressive ability to gather and identify rare specimens along the English coastline. Swainson and Bentinck would warmly correspond from their meeting in 1782 to her death in 1785, often making each other gifts of rare specimens (Tobin 2014, pp. 41, 83, 251–253).^5^ But the greater gift for the young customs officer was undoubtedly his numerous invitations to Margate, Bentinck’s estate and specimen-gallery in Kent, which counted men like Sir Joseph Banks and Daniel Solander (one of Linnaeus’s famed “apostles,” then working as a leading curator at the British Museum) as visitors. It was seemingly here that Swainson met the Linnean Society’s future founder and President, Sir James Edward Smith, with whom he forged a friendship that ultimately led to his unlikely position as a founding Fellow of the Linnean Society in 1788.^6^

William, evidently, learned by his father’s example. During his travels, first as a civilian officer at the British commissariat in Italy from 1806 to 1814, and later on a year-long trip to Brazil, the younger Swainson routinely spent his free time undertaking botanical and zoological campaigns through the surrounding countryside, amassing a significant private collection of specimens rare to Britain in the process. Swainson’s growing collection, however, was not the only key to the social mobility he would enjoy in the years before and after 1820. His strategy, rather, was to make (often-unrequested) gifts of rare and desirable specimens to England’s leading naturalists. Throughout 1816, Swainson sent letters to Alexander Macleay, referencing attached parcels of plants and insects both for Macleay and for other Fellows of the Linnean Society, of which Macleay at the time was Secretary.^7^ Earlier that same year, Swainson had corresponded with James Edward Smith for the first time, whom he had evidently sent a box of rare orchids, and who (quite belatedly) sent him his “best thanks for this interesting present.”^8^ Swainson would be elected a Fellow of the Linnean Society by the end of that year. Like his father, William Swainson saw the value that undertaking collecting work for powerful patrons carried, and similarly came to reap the rewards.

At the same time that it was attracting powerful sources of support, Macleay’s theory rapidly generated a contingent of detractors—even amongst members of the Zoological Club, which was itself designed to attract naturalists seeking to push the field in new directions. Of these quinarian skeptics, two rough groups can be identified: those who cast doubts, like Smith and William Roscoe before them, about the viability of any natural system of classification, and those who advanced rival sorts of natural systems that substantially differed in structure. Of the latter category, one novel natural system, developed in the mid-1820s, came to dominate the discussion. As the decade went on, Adrian Hardy Haworth’s “dichotomous” system was increasingly regarded as the most prominent alternative to Macleay’s quinary theory. Outside these two emergent challengers—one systematically conservative, the other liberal but with differing principles—the quinary theory faced significant internal difficulties. Macleay left London in 1825 to take up a colonial post in Havana, where he would remain for over a decade. While he continued to engage with England’s naturalists in print, his capacity to advance his work through the business of learned societies, be it their creation, their management, or their proceedings, was substantially diminished. Moreover, in his absence, Vigors and Swainson would bitterly end their personal and scientific relations in 1825 over differences of opinion in how to interpret the quinary theory. Half a decade after Macleay had published England’s most discussed work of natural history, and worked to found an institution in which it might be discussed, his theory found itself simultaneously besieged by two camps of detractors and fractured by discord and distance amongst its adherents.

## Haworth, Fleming, and the Dichotomists

**Fig. 1:**
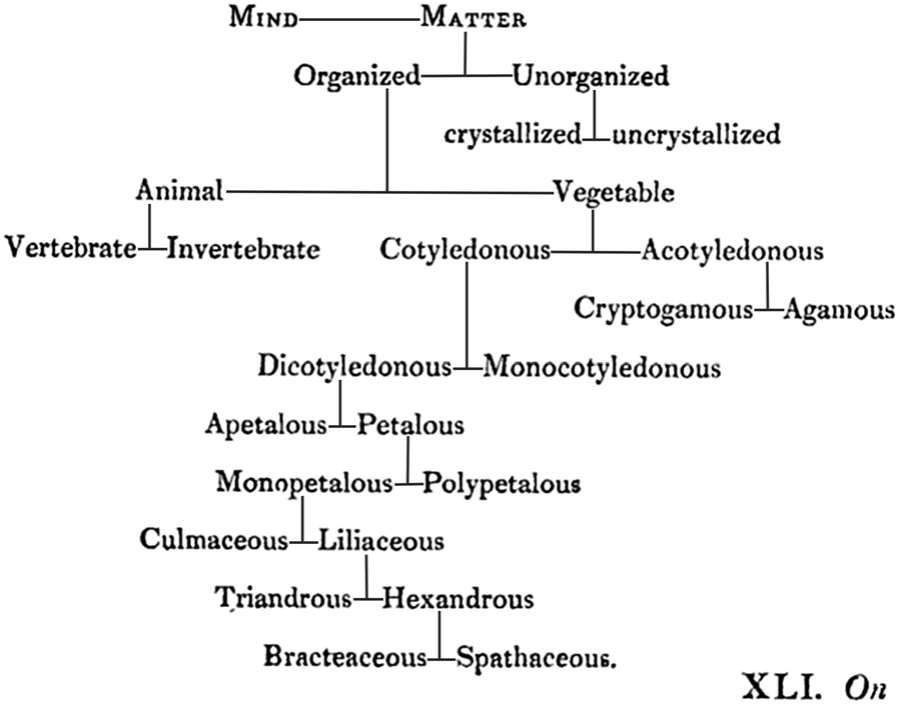
Haworth’s binary arrangement, with an emphasis on his area of expertise—botany (Haworth 1823, p. 202). While Haworth would extend his system into the animal kingdom later in the 1820s, no records exist of him attempting to dichotomously subdivide “mind” into its components

First developed in botany—Haworth’s primary area of expertise—the dichotomous system was subsequently extended into zoology by the Scottish minister John Fleming in the late 1820s. Sources indicate that Haworth first developed his system in 1823, at the late age of fifty-six, shortly after reading Elias Magnus Fries’s *Systema mycologicum* (Haworth 1823, p. 202). Like Fries, Haworth’s basic premise was that all natural groups resolve into a binary arrangement, typically with one member bearing a defining trait, and the other lacking it. For example, animals possessed animation and plants did not; vertebrates bore their namesake in contrast to invertebrates. In other cases, other oppositions could separate the two members of a group—monocotyledons possessed a single embryonic leaf within their seeds and dicotyledons had two. Haworth, however, eschewed Fries’s commitment to “double–dichotomy,” in which binary groups resolved further into a quaternary arrangement when their two original members were themselves shown to bear a dichotomous pair. And unlike Fries’s circular groups, which radiated from a central point, Haworth’s arrangement proceeded from the top-down, beginning with the most “primaeval” division, that between mind and matter, after which the latter group then resolved into matter which was “organized” (i.e. living), and that which was not (mineral). Haworth, it must be said, was not the first Englishman to propose a dichotomous or “bifurcating” system of classification, though he was the first to attempt its extensive transposition onto natural history. As Gordon McOuat relates, the political philosopher and social reformer Jeremy Bentham—Haworth’s contemporary—had recently advocated for a sweeping reform of both logic and law along the principles of dichotomous logic in his 1817 *Chrestomathia* (McOuat 2009). And while Bentham himself attributed the method to the work of French logician Peter Ramus in the early 16th century, the practice of dichotomous analysis extends back centuries to men like Porphyry of Tyre and Aristotle (Fig. 1).

Curiously, despite the popularity of the method, and though he had been a Fellow of the Linnean Society for twenty-five years, Haworth published his first article on the dichotomous system anonymously. It is not immediately clear why he elected to do this. The strongest possibility is that Haworth did not want to publicly contradict Macleay’s theory, to whom he paid regular tribute throughout the article, often stating as fact that natural groups had been convincingly shown to contain five members in many realms of life. Haworth even initially took pains to make his dichotomous arrangement congruent with Macleay’s quinary one, citing Macleay’s bifurcation of normal and aberrant groups-within-groups as a key point of convergence.^9^ But the unavoidable implication of Haworth’s work was that it advanced a radically different arrangement of nature than Macleay’s. Binary divisions could not form circles, and two was not five. Haworth understood this; one advantage of publishing the first suggestion of his system anonymously was that he could closely observe its reception without the need to defend his work. Haworth, furthermore, had no ultimate answer to the fundamental incompatibility of his system with Macleay’s, sheepishly remarking in the final passage of his essay that “[o]f the vegetable and animal groups, each of which are so often and so repetitively divisible into *fives*, forming circles naturally returning into themselves, the writer has not at present leisure to consider…” (Haworth 1823, p. 202). But he would not distance himself from his system for long; by 1825, Haworth was vigorously publishing dichotomous arrangements of different classes of the plant and animal kingdoms under his own name.

Still, Haworth made great efforts to synthesize his system with Macleay’s where possible. In 1825, he wrote that his dichotomous groups “may be imagined (by bending its ends together) as forming a sort of circle of affinity, either open at top, or closed by the root which produces it” (Haworth 1825, p. 429). The language of circles and affinities are a clear reference to Macleay’s work. And like Macleay, Haworth maintained that his system was continuous in its arrangement, forming “as it were, an inverted branching and exuberant tree,” in which each individual branch forked recursively until its ultimate termination at the rank of species (Haworth 1825, p. 429). The central difference, then, remained Haworth’s insistence on two members for each group, as opposed to Macleay’s five. As described above, even this could be reconciled via a liberal interpretation of Macleay’s “normal” and “aberrant” subcategories within each circle. Haworth, however, seems to be the only English naturalist who worked seriously to hybridize his dichotomous system with Macleay’s quinary one; Macleay himself made no documented attempt to produce a synthetic interpretation of Haworth’s system and his own, as he had with Fries in 1823 (Macleay 1823). The majority of their British colleagues who shared their aspirations for discovering the natural system of classification were left to either choose between them, to develop their own, or to look abroad.

By 1828, publications indicate that the dichotomous system had generated a significant local following, rivaling that of Macleay’s. Most notably, John Fleming’s popular *History of British Animals* had rigorously followed a dichotomous arrangement, the inspiration for which he attributed specifically to Haworth. In his introductory remarks, Fleming also took the opportunity to attack Macleay’s quinary system, claiming that it had fundamentally “originated in metaphysical prejudices,” in stark opposition to the close empirical work he associated with Haworth (Fleming 1828, p. xxi). This escalation of tensions was mirrored in the other camp of natural systematists—that same year, the orientalist Henry Thomas Colebrooke published an article for *The Zoological Journal* “On Dichotomous and Quinary Arrangements in Natural History,” in which he commented on the growing rift and subsequently threw his support behind Macleay. For Colebrooke, the dichotomous system was hopelessly oversimplified, ultimately constituting nothing more than an “instructive key to natural knowledge” (Colebrooke 1828, p. 43).^10^ Such invocations of pedagogical utility were a common way for naturalists to dismiss the continued relevance of any system of classification among experts, without actively disparaging the system itself. To claim that a system still possessed instructive merit was to implicitly suggest that it was the purview of students and amateurs. Colebrooke—a distinguished Sanskrit scholar with an avid interest in Indian philosophical systems—felt that Macleay’s, by contrast, was the clear choice for experts seeking to study natural categories more thoroughly.

While Haworth and Macleay respected each other—rarely registering any sentiments (negative or otherwise) about their different systematic opinions on paper—adherents to their respective systems did not always exhibit such circumspection. Among his quinarian allies, Macleay counted Vigors, Swainson, Colebrooke, and John Edward Gray, future Keeper of Zoology at the British Museum, who proposed quinary arrangements of the genera of reptiles and barnacles for the *Annals of Philosophy* in 1825 (Gray 1825a, b). Haworth found his most ardent supporter not in the Linnean, but the Royal Society—in the figure of the aforementioned Reverend John Fleming, a minister of the Free Church of Scotland and graduate of the University of Edinburgh. While Macleay proved reticent to attack Haworth, a fellow founder of the Zoological Club and twenty-five years his elder, he would himself instigate a bitter public controversy with Fleming in 1830 on the subject of his dichotomous arrangement.

## Bicheno and the Nominalists

Not all of London’s younger systematists shared the reformist sentiments of Macleay, Vigors, Swainson, Haworth, and Fleming; certain among them took up the cautionary arguments historically leveled by the most senior members of the Linnean Society. The most notable representative of this perspective was James Ebenezer Bicheno, who in 1825 succeeded Alexander Macleay as Secretary of the Linnean Society after the latter was appointed to a colonial post in Australia. Bicheno hailed a younger generation of the Society’s leadership; at 40, he was nearly a full twenty years younger than the elder Macleay at the time of the latter’s resignation and emigration. His membership to—and eventual presidency of—the Zoological Club also marked a turn in the organization’s character; Bicheno largely followed older naturalists like William Roscoe and James Edward Smith in casting doubts upon the possibility of ever truly uncovering the natural system of classification. One document best exemplifies Bicheno’s sentiments on the matter, his essay “On Systems and Methods in Natural History,” first read at a meeting of the Club on June 4th, 1826, and later published in the Club’s *Zoological Journal* in 1827. Bicheno professed that his comments were occasioned by the jointly-rising popularities of the dichotomous and quinary systems, making general observations on the philosophy of systematics, “a subject of great importance at all times,” “especially so at present, when new views of arrangement and nomenclature are proposed, and to some extent adopted” (Bicheno 1827, p. 479). Foreshadowing his critical remarks, Bicheno also took care to clarify that he was not wading into the ongoing debate in natural systematics: “[l]et me not be understood… to be opposed to any particular system; my object being to discuss the first principles of arrangement, and to leave others to judge how far they are applicable to the views adopted by any individual systematist” (Bicheno 1827, p. 479). He would shortly make it evident that his opposition was indeed not directed at any particular system, but at all natural systems in general, quinary, dichotomous, or otherwise.

Bicheno’s argument proceeded thusly. Due to the recent “readiness with which new systems are adopted,” and considering that in Bicheno’s estimation the “difficulties of the subject have not been duly appreciated,” some remarks on the challenges and pitfalls involved in creating novel systems of classification were warranted (Bicheno 1827, p. 479). Bicheno’s stance was clear from the start;—such systems at present lacked a “philosophical solidity,” threatening the rigor of the science. His suggestion, however, was not to merely return to the consciously artificial system of Linnaeus, as Roscoe and Smith had suggested a decade earlier. Bicheno observed that:

In some respects, it is not to be regretted that the absolute sway which the name of Linnaeus has had among English naturalists is somewhat abated: for although authority is an extremely useful bond of union… yet it has brought with it the ordinary evils attendant upon great names. The range of the pupil has been limited by that of the master; and it has been considered a species of heterodoxy to dissent from the established opinions. The danger to be now appreciated is, that those who adopt other arrangements will forget the advantages to be derived from what is old, in their love of that which is new. (Bicheno 1827, p. 481)

Bicheno thus represented a more moderate form of the systematic conservatism advanced by men like James Edward Smith and William Roscoe in the 1810s (e.g. Roscoe 1810; Smith 1819, discussed in Scharf 2009, pp. 102-107). 11 He readily acknowledged that English systematics had indeed suppressed the spread of new ideas—“it has been considered a species of heterodoxy to dissent from the established options”—but simultaneously adopted a sort of Burkean conservatism, remarkably apparent in the phrase “those who adopt other arrangements will forget the advantages to be derived from what is old, their love of that which is new.” His suggestion, in light of these unfavorable extremes, was on the surface to proceed with extreme caution in advancing new systems of classification. But in actuality, Bicheno encouraged his peers to abandon their efforts entirely. Terms like “species,” “genus,” and “class,” for Bicheno, were entirely heuristic; they amounted to nothing more than vague hypotheses about how living forms might be connected. Though certain naturalists disguised them in the language of mathematical certainty and geometric proof, their position was completely untenable. In taking this position, Bicheno joined the ranks of contemporary conservative scholars like William Whewell, who, as Gordon McOuat has shown, rejected the reducibility of concepts to strict and comprehensive definitions, in part to counteract Jeremy Bentham’s ongoing efforts to capaciously reform law and philosophy along such lines (McOuat 2009, pp. 218–220). Bicheno essentially argued that the natural systematists had merely convinced themselves that working conveniences of signification—terms assigned to vague impressions, a necessary infrastructure of man’s limited faculties—had become natural objects they believed to have found in the external world. And while lower-ranking taxa (species and genus, primarily) approximated truth-to-nature more closely by virtue of the exactness and minuteness of observations involved in their constitution, higher-order taxa like order and class involved so many component assumptions that they were resolutely artificial. Such terms should not be abandoned in light of these truths, Bicheno argued, but should rather be used with clear-eyed and explicit recognition of their fundamentally practical and imperfect nature.

These stances, if adopted, had damning implications for natural systematics. The uncovering of true natural order relied on a confidence in the capacity of the mind to identify actual categories and relationships in-the-world; if that confidence was ultimately misplaced, the entire enterprise was doomed from the start. Evidence of this ailment among natural systems, for Bicheno, was readily apparent. Even if one looked at even the most esteemed natural systems, those produced by Jussieu, Latreille, and Cuvier, all had required the introduction of a multitude of new categories, each founded upon the same rotten foundations as the others—terms like “sub-class,” “cohort,” “tribe,” “stripe,” “sub-genus,” and even “sub-species.” These new categories, for Bicheno, “must be looked on with suspicion” and amounted to no more than “unphilosophical language” that he and his fellow naturalists “deceive ourselves by fancying that we can deal with these delicate and fleeting instruments of thought differently from the rest of the world” (Bicheno 1827, p. 495). The limitations of mind and language made the natural system no more than a flight of fancy, as it would “never submit to be bound by any fetters which the art of man can invent. Books after all are but a rude mode of holding knowledge together; and language but an imperfect vehicle to convey with precision the just relation of things” (p. 496). Bicheno’s nominalist epistemology rendered the pursuit of anything but an avowedly artificial system of classification essentially worthless. Its ambitions were resolutely conservative—to discourage the radical reform of taxonomic categories, and to espouse the enduring value of the Linnaean groupings in use.

Macleay was not immediately present to dispute Bicheno’s claims. In 1825, he had been appointed to the mixed British and Spanish court for the abolition of the slave trade as a commissioner of arbitration, necessitating his move to Havana, where he would remain for over a decade. Nonetheless, he continued to engage regularly with English natural history from abroad, receiving periodicals and sending his own work for publication. His appraisal of Bicheno’s essay, discussed at length below, was not a positive one. Bicheno’s recent chairship of the Zoological Club—the organization Macleay had designed in part to oppose the conservative Linneans just before his departure—undoubtedly intensified its effect on him. Understanding the acerbity of Macleay’s response, however, requires an examination of the two men’s joint history at the Zoological Club.

There is ample evidence of interpersonal challenges between Bicheno and Macleay that well preceded the distribution of Bicheno’s “On Systems and Methods,” though much of it is veiled within the characteristically euphemized language of the Club’s formal records. Minutes of a meeting of the Club on May 25th, 1824 indicate a debate between Macleay and Bicheno, after Bicheno read an apparently critical paper “on the quinary distribution of nature,” after which “[a] discussion subsequently arose on the subject in which Mr. W.S. Macleay, Mr. Vigors, and Mr. Bicheno took part; the continuation of which was adjourned until the next Meeting.”^12^ However, Bicheno did not attend the subsequent meeting on June 8th, and the discussion was “further postponed until the ensuing meeting.” At the next meeting on June 22nd, Bicheno attended but Macleay did not; the discussion “was again postponed.” On July 13th, Macleay was present, Bicheno was not, and the subject was dropped from the minutes entirely. Bicheno’s paper was never published in the Club’s freshly-minted periodical, *The Zoological Journal*. Other factors may have been at play here, but the public hostility exhibited by the two men later in the decade suggests a longer history of contention.

Indeed, the quinary system, when referenced in the Club’s minutes, regularly inspired such “discussions” amongst the members, which were regularly “postponed” until the subsequent meeting. Such was the case on November 23rd, 1824, when Vigors defended Macleay in a debate “on the subject of the quinary arrangement of nature and the circular succession of affinities among the groups of Nature.”^13^ The subject was dropped during the Club’s annual elections on November 29th, but the following meeting on December 14th, in which Vigors read a paper advancing a quinary arrangement of birds, similarly ended with a “discussion” on the “subject of the quinary distribution of nature.” The meeting of January 11th, 1825 ended with a similar discussion, this one, in the words of Secretary James Francis Stephens, “lengthened.” On April 28th, any discussion to follow Vigors’s presentation of a diagram on the tribes and families of perching birds was preemptively “postponed to a subsequent meeting.” The subject was dropped from the next meeting on May 10th, but on June 14th, Vigors spoke at length defending the quinary system against “some objections which had been brought against the principles which had been explained to the Club on this and preceding evenings,” which was again followed by a “lengthened discussion,” and subsequently, “further postponed to a future opportunity.” Such discussions abated in late 1825, when Macleay departed London to take up his colonial post in Havana. However, tensions apparently flared again on March 28th, 1826, after Bicheno presented some “[o]bservations on the ends proposed in Natural History by the use of Artificial and Natural Methods,” which, as had become tradition at the Club, was immediately followed by a discussion, which was, itself, postponed. The following two meetings, on April 11th and 25th, were almost entirely constituted of “conversation” between Vigors and Bicheno on the subject of his paper. On June 4th, Bicheno presented his final rebuttal, an essay “On Systems and Methods in Natural History.” This reached Macleay in 1827, after it was published in *The Transactions of the Linnean Society*. Macleay, it would turn out, did not take it well.

## A Quinarian Rift

At the same time that Haworth, Fleming, and Bicheno’s ideas were attracting the attention of Britain’s naturalists, the internal coherence of the pro-Macleay faction in London was dealt a significant blow when relations between the two most staunch supporters of the quinary system—outside its creator—rapidly deteriorated in 1825. Nicholas Aylward Vigors and William Swainson seem to have become good friends after Swainson joined the Zoological Club in 1824, no doubt spurred in part by their mutual admiration of Macleay’s system. But their correspondence indicates the emergence of a growing rift between them sometime in 1825, when Vigors made numerous references to a series of heated conversations in two letters merely dated “Friday morning” and “Sunday night.” The cause of the dispute seems to have been a difference of opinion in constructing their respective quinary arrangements of birds.

The specifics of their disagreement centered on the precise sequence of the five genera each purported to make up the family *Sylviidae*—the songbirds. Vigors asserted the order to be 1. *Tyrannus,* 2. *Edolius,* 3. *Lanius,* 4. *Thamnophilus* 5. *Ceblepyris*; Swainson held that the proper sequence was 1. *Tyrannus*, 2. *Thamnophilus*, 3. *Lanius*, 4. *Edolius*, 5. *Ceblepyris*.^14^ Why was such a seemingly minute distinction such a serious issue? At stake were two key issues. Firstly, Vigors was about to publish his arrangement in a long paper for the *Transactions of the Linnean Society*, which would directly contradict previously published material by Swainson. The prospect of entering conflicting opinions into publication, even among close friends, could easily create tension. Swainson, perhaps keenly aware of his humble Liverpudlian origins, routinely defended his systematic opinions fiercely, even against men from far wealthier families—men like Vigors. The second issue, however, concerned the quinarian system more directly. In short, Swainson had come to amend various elements of Macleay’s theory by 1825, a trend that would continue and intensify into the 1830s (e.g. Swainson 1835). Vigors was not pleased with Swainson’s liberal reforms, and routinely tacked remarkably close to Macleay’s stated principles in print and in person. He warned Swainson in his second letter that:

There must be great caution used in broaching any principles at variance with the views which we wish to support: & your collateral affinities, or the assertion that two neighbouring groups are allied to each other by equal degrees of affinity at two points, directly militates against Mr. Macleay’s doctrine that neighbouring series touch each other only at one point, & that all other relations are only relations of analogy – I wish I could have a few minutes conversation on this point with you, as this variance of views would at once be seized upon as an insurmountable objection to the justness of our general principles.^15^

**Fig. 2:**
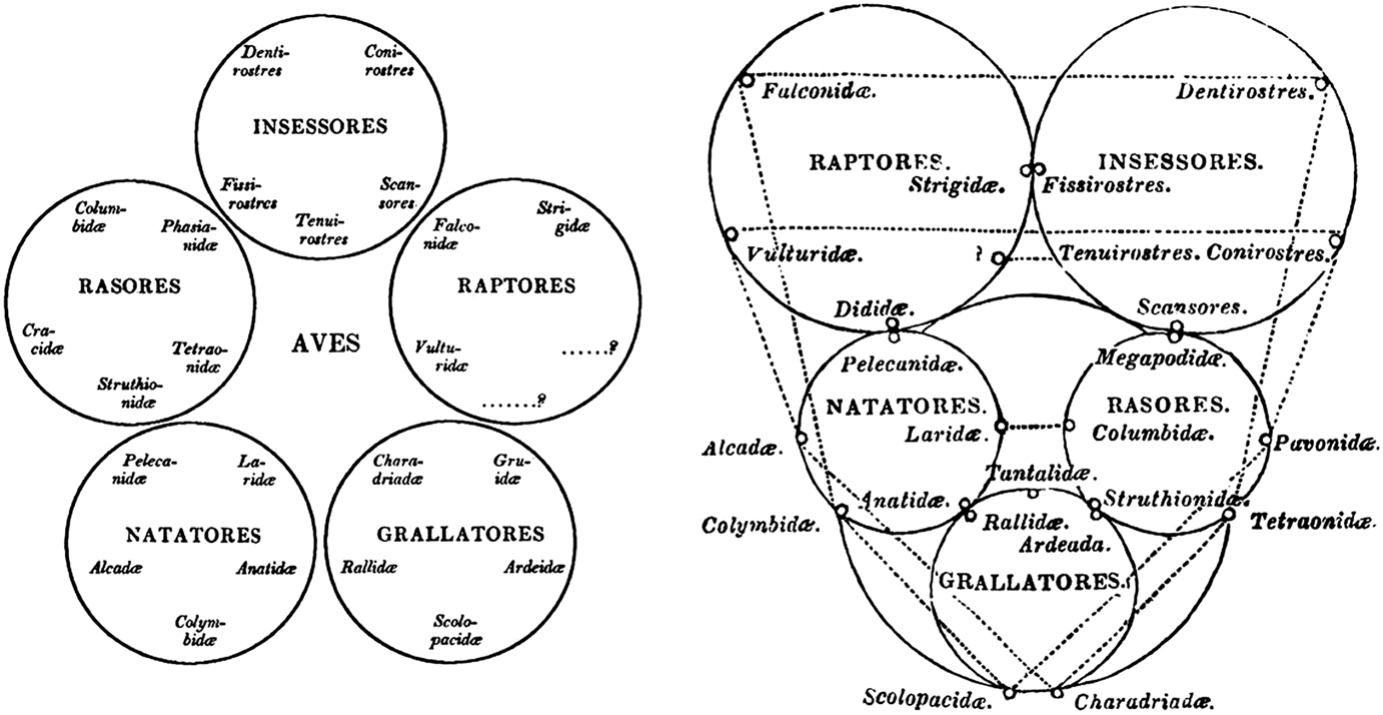
Vigors’s circular and quinary arrangement of the class *Aves* in 1825 (left) and Swainson’s, from 1837 (right). While similar in many respects, the sequencing of orders differs, and Swainson’s diagram further emphasizes the analogical relations between groups, represented by the dotted lines. His “collateral affinities,” however, are not registered in this particular representation (Vigors 1825, p. 509; Swainson 1837, p. 200)

Vigors was right; Swainson’s “collateral affinities,” in which quinary groups were connected at not one, but two points, foundationally undermined the internal coherence of the principles undergirding Macleay’s system (Fig. 2).^16^

Swainson seems to have repeatedly rebuffed Vigors’s attempts at amelioration—this was the last documented letter Vigors ever sent to Swainson. Indeed, Swainson appears to have had a somewhat prickly and easily-offended personality—the early minutes of the Zoological Club indicates that he initially refused an invitation to membership on the grounds that he wasn’t asked to join earlier, when the Club was first formed.^17^ This trend would continue into the 1830s, when Swainson became embroiled in a litany of interpersonal controversies, few of which he proved ultimately reticent to engage in. One of his most notable textual combatants would be none other than Vigors.

## Storms on the Horizon and the Fall of the Zoological Club

The controversy between William Sharp Macleay and James Ebenezer Bicheno, which had its seeds in meeting of The Zoological Club in 1824 and 1825, and spilled onto the pages of the *Transactions of the Linnean Society* and the *Zoological Journal* in 1827 and 1829, would prove a mere prelude to the myriad controversies that would wrack the community of London’s naturalists in the 1830s, drawing notable amounts of public attention in the process. What began in the rooms of the Linnean Society on Soho Square, and was subsequently furthered in the pages of its two periodicals, would soon move into some of London’s most widely-circulated periodicals like the *Quarterly Review, The Philosophical Magazine,* and the *Magazine of Natural History*. In 1830, shortly after the unexpected death of Adrian Hardy Haworth, Macleay would go on the offensive against the Reverend John Fleming in a series of inflammatory pieces harshly criticizing both the man and the dichotomous system of classification. That same year, he would privately spar with his longtime friend and mentor William Kirby, simultaneously ending their long correspondence and, by all appearances, their decades-long relationship. Throughout 1831, Nicholas Aylward Vigors and William Swainson would attack one another fiercely in the pages of the *Magazine of Natural History*, although both men continued to adhere to the quinary system. Later in the 1830s, Swainson himself would take on a myriad of his fellow English naturalists as he felt himself increasingly besieged by London’s scientific elite.

Topics and harsh feelings first broached in private letters and in the exclusive rooms of the Linnean Society would soon become the talk of many of England’s learned circles. The emergence of new theories of natural classification, in the wake of the weakening hold of the Linnaean system on the minds of Britain's naturalists, would not just cause discord in the nomenclature and theoretical literature, as men like James Edward Smith and William Roscoe feared; it would come to serve as the field of play in which a staggering number of acerbic interpersonal controversies would erupt. As for the fate of the organization in which many of these disputes originated, no single event or moment effected the ultimate dissolution of the Zoological Club of the Linnean Society in 1830, just seven years after its inception. Rather, as Adrian Desmond argues, the Club collapsed as a result of two interconnected ailments—diminishing financial resources and declining attendance (Desmond 1985b, pp. 223–224). While the Club was given free access to the rooms of the Linnean Society, its periodical, *The Zoological Journal*, demanded regular payments to W. Philips, who printed it at George Yard on Lombard Street. These funds had traditionally been volunteered by the Club’s members, but as participation waned towards the end of the 1820s, the Club fell into debt, as Desmond describes.

The causes of diminishing attendance at the Club’s bi-weekly meetings are more challenging to unravel, but two likely culprits present themselves. The first was Nicholas Aylward Vigors’s increasing attention to a parallel organization—the Zoological Society of London, which Desmond designates the spiritual successor of the Club and the central drain on its membership. Though Vigors continued to attend meetings of both organizations after the Zoological Society’s foundation in 1826, it is clear that his focus (and his ambitions) increasingly were diverted towards the Society. This was compounded by William Sharp Macleay’s long-term departure from England to his colonial post in Havana in that same year.^18^

The second likely cause of diminished enthusiasm for the Zoological Club is precisely the deepening methodological divides that this article has so far examined. By 1828, the Club was divided into at least three conceptual factions—those in favor of some form of quinary arrangement of nature, following Macleay, those who followed Adrian Hardy Haworth in favoring a dichotomous arrangement, and those best represented by James Ebenezer Bicheno in casting doubt on the viability of any natural system of classification whatsoever. Meeting after meeting, and in volume after volume of the *Zoological Journal*, these conceptual divides routinely showed themselves to be unbridgeable. Macleay and Bicheno’s long-standing rivalry, which reached a head with Macleay’s public letter to the latter in 1829, was both a symptom of the Club’s ailment, and an ultimate cause of its waning momentum in the final years of the decade. It would also serve to foreshadow the outbreak of the numerous controversies between London’s leading zoologists that were shortly to come.

## The Fracturing of Quinary Systematics and the “Decline of Science”

In 1830, all three major proponents of the quinary theory—William Sharp Macleay, Nicholas Aylward Vigors, and William Swainson—enjoyed esteemed reputations as naturalists; by 1840, two of the three men would leave England permanently, their reputations in tatters, and one would be dead. Macleay left London for New South Wales in 1839, just three years after returning from Havana, his legal work in dismantling the local Anglo-Spanish slave trade concluded, and his frustrations with England’s naturalists high. Swainson departed St. Albans in 1840 for New Zealand after a decade of withering public attacks on his character and a near-endless stream of aggressive publication deadlines as he struggled to support himself and his children. Vigors, who had towards the latter half of the decade increasingly eschewed zoological pursuits in favor of a political career representing his native County Carlow, died at home of an unspecified illness that same year. While a select few adherents remained committed to the quinary system across the country, none published or organized in support of it in any volume comparable to the three departed men. Enthusiasm for the quinary system would not remain in England much longer than they did.

Macleay’s system was not destined to be entangled in such fierce controversies, but its conceptual and social environment nonetheless worked in tandem to increase its chances in particular ways. Its dramatic fall from popularity by 1840 was a joint product of its impact on the conceptual landscape of British systematics at the time—consensus about how to classify living things (and to what extent they could be classified), how to describe them and assign them characters, and how to name them—and a consequence of social dynamics in how its contingent of adherents individually elected to characterize and defend it from its critics, and in the cultural and institutional landscape in which they did so, at a time when Charles Babbage and his famous “declinists” had spurred an active national conversation about the just management of scientific institutions and the appropriate means of engaging in debate (Babbage 1830). The end of quinary systematics’ short life in England is not a story of heroic empirical naturalists rooting out numerological evils from their field, it is a story of a field in conceptual, social, and institutional turmoil, and the winding paths its members of varying commitments took in charting their way through it.

Macleay, Vigors, and Swainson’s myriad controversies, furthered in their aspirations to defend and grow the theoretical apparatus that their names and reputations had become so intimately attached to, closely coincided with broader tumults in England’s scientific community. On April 29th, 1830, Charles Babbage published his *Reflections on the Decline of Science in England, and on Some of its Causes*, almost immediately igniting debates about the management of the Royal Society of London, and about the health and status of English science more broadly. At the same time, England was experiencing its most tumultuous political moment in recent memory, which historian Boyd Hilton has called “the crisis of the old order,” characterized jointly by “ideological confrontation” and “political confusion” as Whigs and Tories alike faced stark internal factionalization that many feared would “shatter the existing party system” (Hilton 2006, p. 372). The remarkable volume and tenor of controversies that wracked British systematics cannot be properly related without some assessment of the local and national context in which they played out.

Charles Babbage’s criticisms of England’s scientific institutions and culture have been well documented by historians like Simon Schaffer, David Philip Miller, and Jack Morell. The occasion for Babbage’s comments was an internal struggle within the Royal Society over the election of its next president after the resignation of Humphry Davy in 1827, who, according to Miller, ended his tenure as a “failed leader” who was fundamentally “unable to reconcile warring interest groups in that community” (Miller 1983, p. 1). Babbage penned the *Reflections* after his friend and colleague John Herschel lost the presidency to the Duke of Sussex, the favored choice of an elite conservative faction who resisted growing calls for the Society’s reform. Babbage’s ambitions, Morrell argues, were to take inspiration from the “successful efforts made by the French governments between 1794 and 1808 to create de novo and sometimes to refurbish an impressive set of scientific institutions of which the Ecole Polytechnique was the enviable pinnacle” (Morrell 1971, p. 185). According to Schaffer, Babbage and Herschel were broadly motivated by a desire to “define a new class of industrial experts who could direct economic policy and seize control from the hereditary nobility and cloistered clergy” (Schaffer 2003, p. 278). Those motivations were to be effected by, among other things, increased government sponsorship of institutions of scientific research, the increased openness of such institutions to members outside what Schaffer calls the “intellectual aristocracy,” and increased tolerance for public scientific debate, which they saw as essential to the progress of scientific knowledge, and believed had been stifled by an unmitigated aristocratic politesse.

Miller cautions that Babbage’s *Reflections* should not be treated as a manifesto representing all advocates for reform in British science; he notes “considerable evidence to support that leading reformers were wary of, in some cases in fundamental disagreement with, Babbage’s diagnosis and proposed remedies” (Miller 1983, p. 46). Still, whatever the reformists’ respective grievances and suggested recourses, it is undeniable that 1830 was a year in which strong sentiments regarding how the English ought to run their scientific institutions and navigate public disagreements were readily aired at an exceptional frequency. It was a time in which the tacit cultural expectations of politesse among Britain’s scientific elite were loosened, and the spirited expression of opinions with serious implications for their community and practice were to be admitted to a degree unprecedented in that century.

The situation on the continent was similarly contentious. In Paris, just two months after the July Revolution, Georges Cuvier—arguably Europe’s most famous living naturalist (rivaled only by Alexander Humboldt) —was engaged in a fierce debate before the Académie des Sciences with Étienne Geoffroy Saint-Hilaire, who had taken Jean-Baptiste Lamarck’s mantle as Cuvier’s greatest intellectual rival following Lamarck’s death in the preceding year. The details of the controversy, which Johann Wolfgang von Goethe famously described in a lecture as “of the highest importance for the science,” and by asserting that “[t]he volcano has come to an eruption; everything is in flames,” has been ably and powerfully related by Toby Appel (quoted from Appel 1987, p. 1). In short, as Appel relates, the debate regarded whether “animal structure ought to be explained primarily by reference to function or by morphological laws” —in other words, whether zoologists should first consider the “form” of their subjects or their “function” in accounting for their origins and interrelations (Appel 1987, p. 2).^19^ Elsewhere, in the Netherlands, Coenraad Jacob Temminck continued to spar on matters of classification with fellow ornithologists Louis Pierre Vieillot and our own Nicholas Aylward Vigors, in a period which Eulàlia Gassó Miracle has appropriately referred to as the “systematics wars” (Miracle 2021). No comprehensive account of the extraordinary eruption of controversies in systematic natural history across Europe around 1830 has yet been advanced, and the subject remains one of the most pressing questions in the history of the 19th century life sciences. The following analysis of the British portion and experience of those tumults aims in part to render such an account more feasible.

## Macleay vs. Bicheno

Bicheno first presented his essay “On Systems and Methods in Natural History” to the Zoological Club in 1826, which, as described above, cast doubts on the viability of any natural system of classification—quinary, dichotomous, or otherwise. For Bicheno, *all* classifications were fundamentally heuristic; they constituted a conceptual infrastructure necessary for the mind and language to function, but in no sense did they access true categories embedded in the order of nature. These commitments stood in direct opposition to the growing numbers of British naturalists desirous of discovering the “natural system” of classification, which would purportedly mirror the plan of creation by representing the true relationships undergirding the remarkable diversity of natural forms. The paper reached Macleay sometime in 1827 or 1828 after its publication in the *Transactions of the Linnean Society*. While Macleay was not mentioned by name in Bicheno’s paper, the implicit references to his work, and the implications of Bicheno’s arguments on his ambitions for the field, must have been immediately apparent.

From his colonial post in Cuba, Macleay took up his pen to defend both his theory and his reputation from Bicheno’s indirect attacks. Macleay’s response—in an article for the Club’s *Zoological Journal* innocently entitled “A Letter to J. E. Bicheno,” represented a significant escalation of the tenor of criticism typically found on the publication’s pages. It is, without a doubt, and by a significant margin, the most inflammatory piece to have appeared on the pages of the *Journal* to date. Had Macleay not played an intimate role in the founding of the Zoological Club and in the creation of its *Journal*, and had he not had friends and allies in leading positions at the Club, it is very possible that the article would never have been published.^20^

Macleay’s register in the article was immediately derisive. He opened with a thinly-veiled accusation that Bicheno had merely and meekly conformed to the dictatorial opinions of the Linnean Society’s president, James Edward Smith, remarking that he had read Bicheno’s paper “with some degree of interest, as it derives no small importance from being, as every word shews, clearly written ‘*ex Cathedrâ*’” (Macleay 1829, p. 401).^21^ Smith, as described above, was a classic conservative systematist who cautioned against the reformation of existing taxonomic categories, and thus resisted new theories and methods in natural classification—preferring instead to adhere as closely to the categories advanced by his Society’s namesake as possible (see McOuat 1996; Scharf 2009). Bicheno’s motivations for such purported kowtowing, Macleay suggested, stemmed from his hopes for advancement within the ranks of the Linnean Society: “I therefore earnestly trust that your labours may not go unrewarded, and that you may obtain all the honour and glory which you promised yourself from the staunch Linneans, by this publication” (Macleay 1829, p. 401). The accusation was, in some ways, personal—Bicheno had replaced Macleay’s own father as secretary of the Society three years prior, and at twenty years his predecessor’s junior, represented a newer generation of the Society’s leadership. As Linnean Fellows of Macleay and Bicheno’s age tended to embrace newer theories and methods of classification more readily, the appointment must have been disappointing to Macleay. Younger men with powerful positions in the Society who shared the elder generation’s reticence for systematic reform portended a continued reluctance for England’s most esteemed group of naturalists to throw its weight behind the sort of work that Macleay wished to promote.

Having dispensed with his attacks on Bicheno’s motivations, Macleay moved to critique Bicheno’s argument for systematic conservatism, quoting a passage in which the latter warned that naturalists might “forget the advantages to be derived from what is old in their love of what is new” (Bicheno 1827, p. 481). It is unlikely that the reference to a Burkean sort of conservatism was unintentional here; Bicheno’s argument was well calibrated to appeal to the gentile audience of the Linnean Society’s *Transactions*. Indeed, Bicheno’s nominalist philosophy of systematics had explicitly conservative entanglements, not just in opposing taxonomic reform within natural history, but, as Gordon McOuat relates, in the broader context of William Whewell’s anti-essentialist resistance to Jeremy Bentham’s liberal political philosophy (McOuat 2009, pp. 213-220). Instead of taking a Benthamite position, however, and advocating for the necessity of change through the recomposition of categories, Macleay argued that in fact “there was never a time when Naturalists paid more attention to the labours of their predecessors, whether ancient or modern, then at present” (Macleay 1829, p. 402). Thus Bicheno, Smith, and his allies were not true conservatives, as they did not really love what is old, but rather only what had “proceeded from the pens of the Swede and his most servile admirers.” He cast his opponent not as a reasonable conservative, desirous of preserving what had survived the test of time, but the absolutist acolyte of an unquestionable continental authority. Simultaneously, Macleay’s characterized his own support for the reform of taxa was not a rash liberalism that brushed aside the learnings of the past, but a measured resistance to the dogmatism of the supporters of a Linnaean hegemony.

Having questioned Bicheno’s motives and challenged his appeals to conservatism, Macleay turned his attention to the specific details of the former’s arguments against natural systematics. The first issue with the essay, Macleay suggested, was its complete lack of reference to empirical cases drawn from the observation of natural forms—Bicheno treated the subject “metaphysically, as a Locke, not as a Linnaeus,” and founded his arguments only on “abstract reasoning” that might have been more prudently been “seasoned… a little more with illustrations drawn from observed facts” (Macleay 1829, p. 403). Of course, given Locke’s eminent status, this was a fact to which “no Naturalist ought to object,” but implication was nevertheless that Bicheno, as a naturalist who could only claim to have “described three species of Orchis, and perhaps twice as many Rushes” lacked the expertise to do otherwise (pp. 403, 409). That lack of familiarity with the subtleties of natural forms, Macleay suggested in turn, had led to Bicheno’s fundamental misunderstanding of the natural system in general. Bicheno’s abstract commitment to nominalism would not withstand a sufficiently expansive study of nature, in the course of which he would realize the central fact—the groups apparent in nature were not heuristic figments of the human intellect, they were real. In fact, through Bicheno’s limited experience, he had already begun the process:

It appears you do not regard genera as merely conventional, but as actually founded in nature as well as species. I likewise consider genera *when properly defined*, to be founded in nature, as I have elsewhere said... I will now, however, go further than you, by stating that the groupes you object to, such as class, order, tribe, cohort, and family, are, when properly defined, just as natural as genera... (Macleay 1829, p. 406)

Furthermore, to Bicheno’s argument that taxa became more vaguely defined as they increased in rank, Macleay countered that “the higher we ascend in the scale, and the more comprehensive our groupes are, we may, in general, be assured, that in the same proportion they are perhaps even more natural.” Evidence to this point, for Macleay, was universally apparent to all reputable naturalists: “who will assert that *Animals* form a less natural groupe than *Vertebrata*?… No one, till now, has ventured to call these classes of Mammalia, Birds and Fishes… 'gratuitous assumptions’” (p. 407).

By admonishing the construction of any new taxonomic categories, and in censuring those “who think it advisable to break up the old genera into new ones,” in Macleay’s estimation Bicheno was arguing that “we must remain stationary… with neither greater or less groupes of species than the genera of Linnaeus and Sir James Smith” (Macleay 1829, p. 407). His classificatory skepticism, in short, served two misguided purposes. First, in supporting a philosophical nominalism that denied the existence of real categories, it deterred other naturalists from contributing to the progress of natural history, for which the thorough and painstaking explication of the natural system, “the one great plan of creation,” was the ultimate telos (p. 404). Second, it was a contemptible attempt to curry favor with the Society’s president and its namesake, by fruitlessly defending what Macleay boldly labeled “the defunct or dying Linnean school of England” (p. 408). It was with reference to this fealty to Smith that Macleay landed his final blow. Though Bicheno had championed the proper English conservative values and criticized French naturalists for their liberalism in forgetting “the advantages to be derived from what is old,” he was in fact advocating for nothing more than an epistemic absolutism maintained by Linnean hegemony; Macleay’s final words assured Bicheno that he and his fellow “obstinate heretics” would “continue to wallow in the mire of natural groupes and subdivisions,” wryly remarking that “[p]ersecution, I fear, only serves to wed these last unfortunate wretches to their guilt, and, moreover, is perfectly useless trouble, inasmuch as we may be sure that the world will swim in the orthodox channel at last” (p. 415). Macleay, in essence, had turned Bicheno from a Burkean systematist into one of Napoleonic absolutism.

## Macleay vs. Fleming

William Sharp Macleay’s vitriolic “Letter to J.E. Bicheno” in early 1829 was only the beginning of a long and acerbic campaign he would undertake in criticizing detractors of his quinary theory of natural classification. His next target, however, would not be Bicheno, Smith, or any of the systematic conservatives, but the proponents of the dichotomous system of classification. It was not towards Haworth, however, that Macleay would direct his criticism (who, by all appearances, Macleay respected as an elder colleague) but John Fleming. In Fleming, Macleay found an opportunity to unreservedly critique the system of classification that stood in most direct rivalry to his own, without directly impugning the reputation of its creator.

In certain respects, Fleming and Macleay were aligned in their ambitions for English natural history. Like Macleay, Fleming had also penned a long essay criticizing Bicheno’s views in “On Systems and Methods.” Fleming, however, elected to publish the essay anonymously, as his mentor Haworth had in his earliest explications of the dichotomous system. But, as with Haworth, the move was largely unsuccessful—many readers immediately attributed the piece to him (on account of its repeated favorable references to dichotomous arrangements) from central figures in the field like Macleay to peripheral ones like George Johnston, who collected specimens for John Edward Gray at the British Museum.^22^

Fleming’s critique adopted a much more moderate tone than Macleay’s. He instead praised Bicheno’s essay as “in general excellent,” only regretting that he had “confined his remarks within such narrow limits, when his sagacity and acquirements qualified him for more extensive generalizations” (Fleming 1829, p. 305). This was little more than an exhibition of the rhetorical due diligence expected of an English gentleman—Fleming immediately proceeded to explain precisely where Bicheno’s argument had gone awry. On the impossibility of producing any truly natural system of classification, Fleming concurred with Bicheno, but the latter’s reverence for the Linnaean system was his most serious mistake. Like Macleay, Fleming felt that the Linnaean system was riddled with numerous inconsistencies, errors, and redundancies, and no sensitivity to the “advantages to be derived from what is old” could redeem it. It ought to be replaced, he argued, with another system, similarly artificial, but not nearly to the same degree as Linnaeus’s. Where Bicheno’s artificial systematics bore close affinity to the conservative nominalism of William Whewell, Fleming’s systematics demonstrated far greater similarity to the work of Jeremy Bentham, who similarly sought to reform extant categories along a dichotomous method (see McOuat 2009, pp. 213–218). Both were artificial, but with disparate aims and political valences.

In countering Bicheno, Fleming explained that his dichotomous method far outstripped the Linnaean system in both its simplicity, and its correspondence with fundamental aspects of cognition.^23^ The dichotomous method, for Fleming, was not just an effective technique of arranging a system of interrelated parts, but “the exhibition of a process of thought universally practised by the human mind,” which, as an artifact of the divine creator, reflected also the logic of creation itself (Fleming 1829, p. 312). Evidence for this universality abounded—Fleming disclaimed “all idea of regarding it as a modern invention, or that Peter Ramus has the merit of its establishment” and cited its origins in “the earliest writings of the world,” Deuteronomy 14, which forbade the consumption of those animals with cloven hooves, and by consequence, permitted it for those that did not (p. 312). Attentive readers could find “equally obvious traces” of the method in Aristotle’s classificatory work, where he often distinguished classes by the appearance or absence of characters. The dichotomous system’s superiority was thus founded not just on the grounds of contemporary philosophy, but was routinely demonstrated throughout history, through its recurrent appearance in both Judeo-Christian tradition and classical philosophy. As a fundamental process of human cognition, it was optimal not just in its elegant arrangement of natural groups themselves, but in its correspondence to the very operations of the human mind, as itself also a product of divine creation. In that sense, for Fleming, his favored system was even more “natural” than Macleay’s.

Though Macleay and Fleming shared a general resentment for James Ebenezer Bicheno, James Edward Smith, William Roscoe, and the systematic conservatives running the Linnean Society, the two differed widely in their respective grievances. Their strongest point of correspondence was in their disfavor for the Linnaean system of classification, and for their contemporaries who strove to preserve its use—often at the expense of other systems. But where Macleay’s system fit natural groups into a series of fractally interlocking circles, with each circle representing a group constituted of precisely five members, Fleming’s proceeded by dividing all natural categories in two, one bearing a defining trait, and the other, lacking it.^24^ Furthermore, while Macleay aspired to uncover the “natural system” of classification (of which he found his quinarian system to be the closest modern approximation), Fleming shared Bicheno’s skepticism that any such natural system could be discovered by the human mind. Rather, his dichotomous system ought to replace the Linnaean by the virtue of its elegance, its intuitiveness, and its simplicity. Macleay, it would turn out, was not willing to put their differences aside; where Fleming might have been an ally in the broader struggle against Linnaean dogmatism in English natural history, he instead saw him, like Bicheno, as another obstacle to the progress of the field to which he had dedicated so much effort.

Macleay first made his opinions on Fleming’s system public with a bold declaration of its imminent collapse, in an 1830 article “On the Dying Struggle of the Dichotomous System.” As with his letter to Bicheno, Macleay wasted little time in commencing with a personal attack. He only knew of Fleming, he claimed via “two or three articles in the Supplement to the Encyclopaedia Britannica,” which “remained a monument of his talent for writing on animals that he not only never saw, but would not even know if he saw them” (Macleay 1830, p. 431). Macleay related that Fleming’s other major publication, his 1825 *Philosophy of Zoology*, contained “nothing new but some miserable plates,” but that he had, at the time of his reading, magnanimously elected to leave “the development of his true merits to time.” This changed when Fleming concluded his review of Bicheno’s “On Systems and Methods” with disparaging remarks on the quinary system:

... when what does this clergyman do, but in the most orthodox spirit of theological hate vent his rage, through the medium of the Quarterly Review, on me, who never so much as thought of him! How he got his article inserted there I know not; but, I suppose, that, trusting to his universally applicable binary system, he knew that editors must be either asleep or awake, and with cautious modesty preferred to catch the King of the Quarterly napping. (Macleay 1830, p. 432)^25^

Macleay’s occasion for such wryly derisive language, he insisted, emerged purely from a duty to defend his character—after Fleming’s critical remarks were printed, he was obligated to “take up the gauntlet he has so foolishly thrown down” (p. 433). The language of the gentlemanly duel is central here; Macleay saw himself not as attacking Fleming, but merely responding as a man of his station was required to when, in Steven Shapin’s famous framing of early English scientific debate, “given the lie” (Shapin 1994).^26^ Macleay, furthermore, defended his apparent outrage by citing numerous instances in which Fleming had either misquoted or misinterpreted his work—errors which, he privately insisted to Swainson, were deliberate.^27^ The majority of the rest of the piece involves successive clarifications of these purported misinterpretations, each accompanied by no small amount of wit at Fleming’s expense. Ultimately, for Macleay, the fatal flaw of any dichotomous arrangement was that groups based solely on the appearance of absence of any single taxonomic character would be inevitably too “comprehensive;” they would necessarily contain far too many members to remain structurally sound, in light of the myriad differences one could draw between their members. It was for this reason that Macleay claimed Adrian Hardy Haworth—a “respectable naturalist”—abandoned the system shortly after its inception, “like every naturalist of sense” (Macleay 1830, p. 437). Indeed, Haworth had not published further on the dichotomous system since 1825. Whether this emerged from a lack of enthusiasm or from a fear of Macleay’s pen is uncertain.

Fleming did not let Macleay’s aggression go without response. In the months after Macleay’s article appeared, Fleming sent a short letter to the editors of the *Philosophical Magazine*, “On Mr. MacLeay’s Abuse of the Dichotomous Method in Natural History.” Fleming’s title, in many respects, was misleading; he made no attempt in the short piece to respond to any of Macleay’s numerous critiques of his system’s principles, or his systematic philosophies in general. Instead, Fleming focused exclusively on the rhetoric Macleay had employed in advancing such critiques, which in his words constituted “the exhibition of a mode of conducting philosophical discussion I had never witnessed before” (Fleming 1830, p. 52). Macleay’s words had not just done an injustice to his character, but to the subject of the methods of classification in natural history, which was “of very great importance to the interests of the science,” and the discussion of which thus deserved to be “conducted in a suitable manner.” Moreover, Macleay’s tenor had necessarily ceased all possibility for what might have been a valuable and productive debate, for “Mr. MacLeay, having laid aside the language of a gentleman, and violated the customary civilities of life, has compelled me, in due regard to my own character, to pass over in silence this effusion of his pen, which is probably without a parallel in the records of science” (p. 52).

Swainson took note of Macleay’s recent behavior, both in his attacks on Bicheno and on Fleming, and wrote a concerned letter in 1830. In response, Macleay thanked Swainson for his “kind advice” and assured his friend that he was “truly sorry” that his “answer to Fleming has not given you satisfaction.”^28^ He cheekily promised, furthermore, that he would “endeavor for the future to be more moderate, in short a good boy.” But despite such reassurances, Macleay immediately noted that his behavior was unlikely to change:

But I must say that this promise of mine is not worth much, for when I see a humbug dishonestly misquoting me to answer his own purposes and [illegible] this humbug to be eared like a horned owl, my blood rises, and the devil laughing at my bows of moderation dips my pen deep into gall. It is all nonsense about Flemings being a clergyman. If he be, there is more reason why he should not be dishonest. Indeed, in flagellations of this kind, a clergyman deserves no better treatment than others, for if he cannot demand personal satisfaction he ought to [illegible] that behavior which cannot be demanded of him and therefore he ought not to give provocation.^29^

For Macleay, it was *Fleming* who had broken far more grievously with the norms of gentlemanly discourse, in mischaracterizing his stated opinions on the quinarian system for his own ends. Macleay’s assessment of the situation gives crucial insight into his estimations of what was warranted as a gentleman of science; it is one of the only documented occasions in which he privately justifies his conduct to a friend. Fleming’s “dishonest” and deliberate misquotations amounted to a serious affront to Macleay’s character, which were exacerbated by Fleming’s status as a clergyman: they denigrated Fleming’s profession and denied Macleay his rightful recourse to “personal satisfaction” —a duel, “that behavior which cannot be demanded of him.” Nonetheless, Macleay shared with Swainson that he could “only hope to be in England within the year,” so that he might stake his life upon his word as a reputable naturalist. Whether he was earnest or not is uncertain; Macleay would not return to England until 1836, and he never sparred publicly or privately with Fleming again, to his friend’s likely relief. Ironically, within a year of his letter, Swainson would have difficulty following his own advice to Macleay, as he became himself embroiled in a series of vitriolic controversies with a fellow naturalist.

Macleay, vitriol aside, made strategic choices in framing his polemics. His attacks on Bicheno’s systematic conservatism and on Fleming’s dichotomous system are explicitly written as personal letters, which were then forwarded to the editors of the periodicals they appeared in. The genre of the private letter provided Macleay with an expanded rhetorical space for expressing harsh sentiments in late Georgian culture; Macleay could claim he was merely expressing his views privately, portions of which editors then found relevant and worthy of presenting to their readership. Whether or not any such personal correspondences were actually sent is doubtful.^30^ In the case of his attack on Fleming, Macleay took this strategy a step further: the article is presented as a personal letter to Nicholas Aylward Vigors on the subject of Fleming, which Vigors himself then recommended to the editors of *The Philosophical Magazine*. But Macleay’s original draft manuscript, entitled “On the Latest Device for Bolstering up the Dichotomous System,” was not formulated as a letter at all, and instead takes the form of an essay. This approach distributed responsibility across three separate parties—Macleay expressed the opinions, Vigors deemed them worthy of the pages of the *Magazine*, and its editors ultimately shared his sentiments. It is possible that Macleay and Vigors learned from the reception of Macleay’s “Letter to J.E. Bicheno” of the preceding year, which took the form of an open letter sent directly to the editorial board of *The Zoological Journal* (of which Vigors was a leading member) and thus read more as a public attack. Their new approach carried with it further rhetorical protections.

Regardless, the readers of *The Philosophical Magazine* took notice of Macleay’s exceptional lack of propriety. In the article’s continuation, printed in a succeeding issue, the editors opened with a note responding to a number of complaints about the piece:

Upon reconsideration of this article, we cannot but regret, in common with many others who take interest in the discussion, that so much personality should have been introduced into a scientific controversy; and Mr. Macleay’s paper having been printed *entire* for private circulation, we have, in acquiescence with the general opinion, omitted, in the continuation which follows, and which will be concluded in the next number, many paragraphs, &c., irrelevant to the subjects discussed. The portions of the paper, therefore, which our readers have now to peruse, must be considered as consisting only of a series of connected extracts from the original; containing, however, all the arguments advanced respecting the dichotomous system. (Macleay 1830, p. 53)

Macleay’s extraordinary expressions of “personality” were ultimately not sufficient warrant to halt publication of the continuations, which had evidently attracted no small amount of attention, and may have stood to boost their subscriptions, as David Lowther argues (Lowther 2020).^31^ Editors were thus presented with a challenge: to attend to the gentile sensibilities of their readers whilst simultaneously allowing space for appropriate amounts of intrigue that would facilitate their sales.

What such “gentile sensibilities” were in 1830, however, is more challenging to precisely characterize. As historian Raf de Bont argues, “19th-century scientific etiquette was not often explicitly codified,” but traces of its expressions remain in works of moral philosophy (de Bont 2013, p. 312). William Paley, a popular professor at Cambridge, felt that controversy ought to be avoided at all costs, which threatened to “put to an end all the comfortable intercourse of our society” (quoted from de Bont 2013, p. 312). De Bont cites the collective insights of Martin Rudwick, James Secord, and Arnold Thackray in asserting that, on the whole, late Georgian naturalists and natural philosophers were “most fearful of controversy” (p. 314). There are reasons to believe, however, that this attitude existed far more in principle than in practice. Works of moral philosophy indeed expressed aspirations for certain norms of gentlemanly discourse, but they are norms that were routinely broken in the print culture of the late Georgian period. Still, that is not to say that controversy was expressly condoned, and that men who discouraged it did not exist. But at the same time, others asserted a role for vigorous debate, like Charles Babbage, who argued in his 1830 *Reflections* that for “[t]hose whose sole object is truth, can have no apprehensions from the severest scrutiny. There are few circumstances which so strongly distinguish the philosopher, as the calmness with which he can reply to criticisms he may think undeservedly severe” (quoted from de Bont 2013, p. 315). The written opinion of moral philosophers should not be taken as a representation of the entire culture they sought to prescribe to. Regardless, Macleay’s acerbic rhetoric certainly crossed a number of culturally-expected lines for a man of his station.

## Macleay’s Retreat

There is evidence to suggest that Macleay’s passion for systematics was not the exclusive cause of the harsh sentiments he so publicly expressed towards fellow naturalists in 1829 and 1830. In June of 1830, Macleay broke a long-standing friendship with entomologist William Kirby, whom he had known since childhood, whom his father counted as his closest friend, and who had helped spur his love of natural history on botanizing and entomological walks through his estate at Barham in the late 1800s (Swainston 1985). The letter from Kirby referencing their argument (and the last recorded letter the two would ever exchange), indicates that Macleay’s grievance regarded his expectation of some unfavorable comments on his systematic opinions, forthcoming in the final volume of Kirby’s *Introduction to Entomology*.

By Kirby’s response, Macleay’s letter had been extraordinarily harsh—had Kirby, then a septuagenarian, “given way to the feelings of irritation produced by the first perusal” of Macleay’s letter, “if indeed it was not intended as a joke,” he “might have employed language, but more justifiably, that would have been equally offensive” to Macleay.^32^ The two men had come into private conflict before—similarly, over their comments on each other’s work in their publications—but this letter constitutes a significant escalation of the register in which they typically discussed such matters. Referencing their contrasting views on natural classification, Kirby in his reply continued by assuring Macleay that the “difference of opinion between us has never resulted from any inimical feeling on my part, but from the different view in which natural objects, in many respects, appear to me.” Such did not, in his estimation, offer Kirby any protections from public rebuke, who welcomed Macleay to publish any critical observations on his forthcoming work. However, he explicitly urged him to do so “in the spirit of a gentleman & a christian which can cause us no uneasings.” Still, Kirby felt Macleay’s extant transgressions demanded some response, and he promised Macleay that in his forthcoming contributions to Dr. Richardson’s *Fauna Boreali-americana*, in which he would “not seldom have occasion to controvert some of your opinions,” some critical remarks would need to be made, though he assured him it would “be done in such a spirit as becomes a Christian Philosopher.” Kirby did not subscribe to many elements of the quinary system, but he only offered comments on it “when the nature of my subject requires it” —in cases where he felt a responsibility to comprehensively survey the different modern systems by which insects were classified. But Macleay’s conduct had now saddled Kirby with a different set of obligations; his duties as an English gentleman demanded that he publish critiques of his old friend’s work where he otherwise might not have. Such was the delicate balance that gentlemen naturalists played when respectively advancing differing systems of classification. Still, for Kirby, such irreconcilable differences of systematic opinion—which were so endemic to British natural history by 1830—were not a sign of illness in the field (as some of his contemporaries argued), but a natural outgrowth of the pursuit of scientific truth. He had no issue with their disagreement, only the rhetorical way Macleay engaged with it. Kirby’s final written words to the naturalist he had first introduced to the wonders of nature were:

You and I are both contending for what seems to us to be the Truth, and the Truth we know will finally prevail; if, therefore, your system is true in all its parts, my opposition to those parts of it in which I differ from you, instead of injuring, will only serve at the long run to establish it; and if it be not true, as far as it is so, it will ultimately die, in spite of all your efforts to uphold it.^33^

Macleay would have agreed, at least in principle; he never claimed that the quinarian system was a perfect representation of the true natural system at which it sought to describe. But by 1830, the bevy of attacks it had weathered in print—some of which he rightly assessed as misinterpretations of his systematic commitments—had evidently left Macleay particularly sensitive to critique. Losing the support of the man who had helped introduce him to natural history as a boy would have stung all the more.

It is undeniable that Macleay’s behavior in 1829 and 1830, expressed through his private and published writings, constituted a significant intensification of his generally-irritable demeanor in comparison to the years prior. Still, caution must be exercised in attributing Macleay’s written expressions exclusively to a petulant disposition in those two years; his critiques were indeed filled with scathing language and personal attacks, but they were also undoubtedly characterized by an impressive array of detailed systematic arguments. Indeed, there were reasons for a natural systematist to be frustrated with the state of his field in 1830, and to think that certain exceptional measures in defending his work was warranted. The Linnean Society continued to promote and be guided by systematic conservatives like James Ebenezer Bicheno and James Edward Smith, who seemed perfectly content for natural classification to remain largely as it had been in 1768, with the publication of the twelfth edition of Carl Linnaeus’s *Systema naturae*. The Society’s Zoological Club, which Macleay had faced such difficulties in founding, faced waning membership and financial instability in his absence, and would ultimately dissolve within the year. And two prominent naturalists, one the standing Secretary of the Linnean Society, and the other a Fellow of the Royal, had recently published serious challenges and critiques of his system of classification, often misrepresenting or mischaracterizing (whether deliberately or not) significant elements of its construction. At best, we can say that Macleay’s aggressive public stances between 1829 and 1830 were the product of two intertwined forces—genuine conviction in his systematic opinions and passion for the science, and growing agitations emergent from both interpersonal and philosophical differences. Regardless of causes, by the end of 1830 he had clearly done significant damage to his public reputation—and by association, to the reputation of the system of classification with which he was so closely associated. Readers had complained; editors had censored subsequent letters. Macleay’s pace of publication slowed dramatically. In a letter to the famed botanist Robert Brown in September of 1832, he shared, with no small amount of shame, that “I am sorry to say I note a sad difference in myself…I fear the truth is I am not the ardent lover of nature’s beauties I once was.”^34^ From a man who had so vociferously defended his natural historical opinions in the prior year, the change was striking indeed.

## Swainson Takes the Mantle

After the tumults of 1829 and 1830, William Sharp Macleay increasingly refrained from public defenses of his work, and from publishing works of natural history in general. Whether this was a product of remorse, exasperation, or disillusionment is uncertain. But Macleay’s retreat from active engagement with British natural history did not constitute a disappearance of the quinarian system from the field. As the 1830s went on, William Swainson—the Liverpudlian son of a customs clerk, renowned illustrator, and Fellow of the Linnean and Royal Societies—would come to take Macleay’s mantle as the primary advocate of the quinary system of classification, and would serve as the most active publisher of work following its principles. But Swainson, too, would quickly come to be embroiled in a series of fierce public controversies, not just with wealthy gentlemen who disparaged his humble origins and critics of his preferred system, but with the quinarian system’s third major adherent, his former friend Nicholas Aylward Vigors.^35^

Swainson’s troubled path through the tumults of the 1830s was, in many respects, unique. Lacking an estate, a college degree, or an alternate profession, Swainson had transformed himself into one of England’s first career naturalists, supporting himself and his family primarily from his publications. From this vantage, ongoing debates surrounding the proclaimed “decline of science” in the country—and broader questions regarding the just organization and management of England's scientific institutions—interested Swainson greatly. Indeed, Swainson wrote to Charles Babbage twice, once in 1832 and again in 1834. His first letter broadly praised the principles and values Babbage had espoused in the *Reflections*, and enclosed the constitutive articles of his controversy with Vigors and the Zoological Society of London, which he suggested Babbage might find useful in his efforts to convince British scientists of the need for institutional reform.^36^ The second requested any supplementary materials Babbage might possess on the subject of such reforms, which Swainson planned to comment on extensively in his forthcoming *Preliminary Discourse on Natural History*. His chief object, he related, was

to shew that none of our Societies are conducted upon principles agreeable to their proposed object and that there exists a necessity for great reforms or for the formation of a distinct Society composed exclusively of men of known and eminent talent, wherein, in short, the Elite of the Science of the country should alone be admitted.^37^

Babbage responded kindly to the first letter but seems to have ignored the second. His reply in 1832 encouraged Swainson to continue his calls for reform in zoology, but indicated that he would not lend Swainson any public support, as it was “better for the cause of science that gentlemen who are themselves strong should apply themselves to their own subjects thus their views will have greater weight.” At the same time, he cautioned the difficulty of such an undertaking, noting that while he did not “regret” his essay—which he “published…without concert with any one and against the advice of several I esteem” —he had “seen above fifty critiques on it,” and far fewer favorable words. Swainson and Babbage, for the time, very much felt members of the minority position.^38^

Understanding Swainson’s views on England’s institutions of science in the early 1830s requires a familiarity with events in Swainson’s career a decade earlier. In 1822, Swainson had applied to be Assistant Keeper of Natural History at the British Museum, when William Elford Leach, its previous occupant, resigned, increasingly incapacitated by an undescribed mental illness. The support that Swainson marshaled for his bid, documented in his application for the post, would have been remarkable for any man—much less the son of a customs clerk from Liverpool, with no Oxbridge background and scarcely any formal schooling to speak of.^39^ Among those who wrote letters in support of Swainson’s nomination were William Jackson Hooker, Regius Professor of Botany at the University of Glasgow and future Director of the Royal Botanic Gardens at Kew, Lockhart Muirhead, also a professor at Glasgow, William Roscoe, elder member of the Linnean Society of London, Thomas Stewart Traill and John Bostock, both professors at the Liverpool Royal Institution, and, at their head, Georges Cuvier, who in his letter described Swainson’s work in the 1821 *Zoological Illustrations* as “meticulously executed in every detail” and which “the public can only gain from knowing of” (Swainson 1822).^40^ Swainson’s impressive roster of supporters, coupled with his recent inductions to the Linnean and Royal Societies, undoubtedly inspired no small amount of confidence that he would succeed in his application. But the assistant keepership was instead given to John George Children, a librarian at the British Museum and friend of Sir Humphry Davy, President of the Royal Society (Miller 1983, p. 44). It was obvious to many that Children had published far less in zoology than Swainson, and the selection was immediately perceived by Swainson and his supporters as a nepotistic appointment of a less qualified man. Upon hearing the news, an outraged Traill wrote to Swainson in April of 1822 that he not only “grieved for the disappointment of a friend” but for the

indescribable injury natural history must sustain by such an appointment… Mr. Children, I am told, is a man of intelligence and great respectability – but alas, he is utterly misfitted for the care of a collection of natural history being ignorant of almost every branch of that important and delightful science.^41^

Traill’s words were not merely conciliatory, meant purely to ease the wounded pride of his friend; for the remainder of the 1820s, he would undertake a written crusade against the Trustees of the British Museum, penning allegations of mismanagement in 1823, 1825, and 1827. Traill’s articles, as David Philip Miller relates, “raised hackles in scientific circles” and even roused the attention of what Traill called “a party of influence within the royal Society” that “would risk every thing sifting the whole to the bottom” (Miller 1983, p. 44). Yet little of these aftershocks yielded Swainson with any personal recompense, and he bore resentment towards the British Museum, and indeed with many of London’s scientific institutions, until his dramatic departure from England in 1840.

Swainson’s views on the state of science in England were also intimately connected with his opinions on, and appraisals of, French science (as was the case with many of his contemporaries), many of which he seems to have developed following a visit to Paris and the *Jardin des Plantes* in the early 1820s. In short, Swainson admired the support for the state sponsorship of science he found in France, and the meritocratic principles its leading institutions espoused. As a man who had risen to his membership from humble beginnings, and whose income relied exclusively from private sales of his scientific works, these positions are intuitively understandable. Swainson made these sentiments public for the first time in March 1831, in response to his former friend and fellow quinarian Vigors’s recent critical piece on French naturalists for *The Zoological Journal*. Swainson’s response, “A Defence of ‘Certain French Naturalists,’” (which borrowed Vigors’s polite framing for its title) instigated a public row between himself and his old friend, with whom his relations, as described above, had rapidly deteriorated in 1825. By contrast, their far more public affair in 1831 serves as a prism through which a number of dynamics are refracted—the tensions that accompanied growing calls for scientific reform, often along a French model, the conservative nationalistic sentiments those calls provoked in turn, the harsh impacts that differing views on emerging theories of classification could have on interpersonal relationships, and the mounting disharmony among supporters of the quinary system more broadly as the movement increasingly lost its internal cohesion.

## Swainson vs. Vigors

David Lowther has written ably on certain elements of Swainson and Vigors’s contentious relationship, focusing primarily on letters exchanged in 1824 and on their public controversy in 1831. Though the two men shared mutual admiration of each other and of Macleay’s quinarian system, Lowther relates that tensions emerged almost immediately when Vigors refused “to put forward Swainson’s name for election to the Zoological Club,” declaring that “[w]e feel a disinclination to urge any man who is not an ardent volunteer in the same cause” (Lowther 2020, p. 118). Lowther attributes Vigors’s “cold ferocity” to Swainson’s “foot-dragging” regarding promised quinarian publications, and his distaste for Swainson’s recent controversy with the British Museum, an institution which Vigors considered central to “our exertions in zoology” (quoted from Lowther 2020, p. 118). While there are good reasons to believe these factors played a role, a more likely and more simple answer is that Swainson had refused an offer to join the Zoological Club in 1823, citing his offense that he had not been invited sooner, and would thus not be considered a founding member.^42^ And contrary to Lowther’s suggestion that the matter “marked the end of their tepid attempt at collaboration,” the two evidently continued to correspond and collaborate until 1825, when their differing opinions on how to interpret the fundamental principles of Macleay’s system proved an unbridgeable rift (Lowther 2020, p. 118). Still, Lowther is right to point out that, already by the mid-1820s, “the Quinarian camp was dividing” and that, irrespective of the specific causes, the two mutually bore some significant animosity by the time Swainson published his critique of Vigors for Scottish botanist John Claudius Loudon’s widely-circulated *Magazine of Natural History* in early 1831. This was no accident—Lowther describes Loudon as an “extremely shrewd and energetic publisher” who had cut his teeth on the *Gardener’s Magazine*, which had enjoyed a notable circulation in the 1820s (Lowther 2020, p. 119). The *Magazine of Natural History*, according to Lowther, sought to navigate “the fine line between ‘popular’ appeal to the interested amateur, and more specialist content that engaged Britain’s growing class of dedicated gentlemen naturalists.” Gordon McOuat describes it somewhat more forcefully—as an “establishment mouthpiece” and “arch enemy” of Neville Wood’s radical middle-class *Naturalist*, the latter of which sought to usurp the epistemic authority of the metropole and effect sweeping reforms of zoological and botanical nomenclature (McOuat 1996, pp. 496–497). Challenging the rising popularity of the *Naturalist* required precisely the sort of deft navigation Lowther sketches—printing content that appealed to London’s gentlemen and provincial workers alike. In pursuit of those ambitions, Loudon increasingly reserved a section of each issue for letters, which Lowther argues constituted “a tactic that was designed to stoke the sort of controversy between naturalists that would keep readers engaged” (Lowther 2020, p. 120). This, in turn, was not a simple product of Loudon’s greed—Lowther notes that the life of a journal in early 19th century Britain was often “short and brutal” as “competition between publishers was intense and editors were unscrupulous in their determination to keep circulation figures high.” Swainson’s article was precisely the sort of piece Loudon hoped to print.

Whether conscious of the connection to his own publisher or not, Swainson’s opening comments lamented the recent state of English zoology—and indeed, English science in general—noting that he and other naturalists “must have observed, with deep regret, the extraordinary mode of conducting scientific discussions which has of late arisen among us” (Swainson 1831a, p. 97). Furthermore, he identified two distinct types of such “extraordinary” publications, the first of which were those that wore “an appearance of being private communications… and as such should not have been published.” This seems to have been an explicit reference to Macleay’s attack on Fleming in the preceding year, which Swainson interestingly (and no doubt, as it suited is purposes) implicitly laid the greater blame on Vigors, who had himself forwarded Macleay’s letter to the editors of *The Philosophical Magazine*. Significant blame also laid with those editors, who Swainson argued did equal harm to all parties involved, as he who deliberately publishes a scathing private correspondence “neither consults the reputation of the writer, nor the taste of the reader; still less does he regard the character of his own journal” (Swainson 1831a, p. 98). Swainson’s framing expressly paints his friend Macleay as a victim of Vigors and the editors’ unjust behavior; whether Swainson knew that Macleay had never intended for his “letter” to Vigors to be kept private is uncertain.

Nonetheless, Swainson’s proclaimed target was a second sort of article, those which “as being avowedly written for the public eye, are much more calculated to foment bitter feeling among individuals, and to bring national reproach upon us all.” In this case, Swainson was specifically referencing Vigors’s 1828 article for *The Zoological Journal*, in which he had defended his quinary arrangement of the family *Psittaciade* (parrots) from some critical remarks by French ornithologist René Lesson in the *Dictionnaire des sciences naturelles*. In the article, Vigors had not just taken exception to Lesson’s systematic opinions, but rather had elected to frame the entire issue as a case of haughty and superior French naturalists dismissing British views on classification on purely nationalistic grounds. Vigors thus claimed he felt compelled to protect the views of his “new school of zoology” from “arbitrary influence which seems exerted to check them in their infancy” (Vigors 1828, pp. 91-92). Indeed, had Lesson’s remarks only regarded his personal opinions, he apparently would not have advanced an article on the subject at all, as “the personal feelings of individuals” were “a subject at all times of little interest.” But according to Vigors, the matter was “rather national than personal,” emergent from a “disposition to depreciate the Zoological labours of this country” that “prevails to a great extent among the Continental writers.” It was against these “mandates of assumed authority” that Vigors responded (Vigors 1828, p. 92).

Though Swainson largely shared Vigors’s enthusiasm for the recent developments in zoological systematics in England, he rejected this nationalistic framing entirely. His article in March of 1831 argued that while Britain had made significant strides in zoology in the past decade, it was “yet still in its infancy,” and while “[w]e have caught a glimpse of some mighty truths, which are not thought perceptible by our neighbors,” there was no cause for such prideful assertions of superiority, cautioning readers that just “because we have discovered a *part* of the natural system, we must not arrogantly imagine that we have grasped the whole” (Swainson 1831a, p. 98). There was no conspiracy among French naturalists against their British colleagues, as Vigors insinuated—they merely “choose to study nature in their own way.” Men who stirred up nationalistic rivalry in a science at such an early stage in its development, furthermore, were behaving like school children; Swainson condescendingly asserted that “our ‘infant school’ may probably be compared to the boys in the story, who got possession of a little puddle, from which they bespattered every passenger who refused to take a *sup*.” As Swainson knew his old friend must have been well aware, the quinary system—which Vigors so proudly identified as a product of the British Isles—took far more inspiration from French natural history than from work in his native land.

The nationalistic and political alignments here become thoroughly entangled. Macleay developed his system after spending four years in Paris, where he engaged with Cuvier, Lamarck, Latreille, and others—men whom he considered to be the greatest naturalists of his time. The quinary system, while different in many respects from the ones respectively advanced by those men, nonetheless took significant inspirations from Cuvier’s comparative anatomical methods, which prized careful analysis of internal structures in deriving taxonomic characters, Lamarck’s insistence on a resolute lack of *salta* or gaps in nature, and Antoine Laurent de Jussieu’s grand ambitions to uncover the natural system. After Macleay returned to London and published an extensive elaboration of his system, it was met with widespread admiration by adherents and skeptics alike as an original, sophisticated, and provocative work of British natural history, one that by all appearances rivaled the work emanating from Paris. Men like Vigors, who had been wounded fighting the *Grande Armée* in the Peninsular War, saw Macleay’s theory as evidence that British natural history had finally emerged from the long shadow of the *Muséum National d’Histoire Naturelle* across the channel. Swainson, whose past friendship with Vigors had deteriorated due, by all extant accounts, to divergent interpretations of Macleay’s work, still generally shared Vigors’s commitment to the theory’s superiority. At the same time, he acknowledged its French roots, and bore increasing admiration for the men and institutions of French natural history in a moment where Charles Babbage had put the “decline” of British science on the lips of many learned gentlemen. Vigors himself had advocated for reform in British zoology in the early 1820s, critiquing an elder generation of British naturalists who had resisted amendments to the Linnaean system of classification as it was expressed in the twelfth edition of the *Systema naturae*, and lending his efforts to form new institutions—The Zoological Club of the Linnean Society and the Zoological Society of London—in which new systematic theories like Macleay’s might be circulated. But by the early 1830s he had adopted a more conservative stance, unwilling to tolerate modifications of the quinary theory and dismissive of many calls to reform London’s scientific institutions, which he now played a far greater role in managing. Swainson, on the other hand, remained a staunch reformist throughout the period, embracing the quinarian system against the Linnean Society’s reticence, modifying the theory where he felt it was warranted, and lending his pen to reformist sentiments in British science. In contrast to Vigors—although both could boast membership to both the Linnean and Royal Societies—he never occupied positions of power within them. And when he had attempted to attain such a post at the British Museum in the early 1820s, he had been passed over in favor of a friend of the Royal Society’s president.

A question remains: Vigors had published his critique in 1828, so, why did Swainson wait for three years to publish a response? While the distaste for Francophobia that Swainson expressed is well aligned with his documented opinions, private letters exchanged between Swainson and his friend, the renowned American ornithologist John James Audubon, reveal an ulterior motive. Swainson and Audubon had visited the Zoological Society—of which Vigors was Secretary—in late 1830, with the intention to visit their rapidly-growing collections. But, for unknown reasons, the two men were denied access to many of the cabinets, and promptly left in outrage (Swainson 1831b, p. 482).^43^ Personal animosity resulting from Vigors and Swainson’s deteriorating relationship, first documented in letters from 1825, is a probable cause—one that Swainson himself subscribed to. Again, vested interests among London’s natural historical elite had denied Swainson a right to which he felt his reputation entitled him. Swainson did not directly mention the affair at the Zoological Society in his initial attack on Vigors, but his frustrations with Vigors’s stirring of Francophobia among Britain’s zoologists align closely with his anger at the seemingly arbitrary authority exercised by the leaders of London’s scientific institutions, considering his favor for the open and meritocratic values of their French counterparts. Vigors defended himself in two subsequent articles, condemning Swainson’s conduct as rash and ungentlemanly in the process, and accusing him of cynically adopting Babbage’s declinist position purely to give credence to his self-interested and unfounded sentiments:

The “decline of science” is the cant of the present day. Every man who has a petty grievance to bring forward, or some trivial point of minor information on which he hopes to be borne into notice, adopts the decline of science as the post from which he starts. (Vigors 1831, p. 335)

The accusations motivated Swainson to relate the entire affair with Audubon at the Zoological Society, registered in a “final Statement…in Reply to Mr. Vigors” in November 1831. In the article, Swainson painted Vigors as a sort of dictator of the Zoological Society, with its founder Stamford Raffles dead, and its founding secretary Thomas Horsfield retired. With Vigors left in sole control, “[t]he whole concern,” Swainson argued, “had assumed the characteristics of any thing but a liberal scientific institution” (Swainson 1831b, p. 482). As evidenced by his denial of access—desired specifically for the purpose of conducting scientific observations—it had become, under Vigors’s leadership, “a society where science was not wanted.”

But Swainson’s “final Statement” to Vigors did not only register his accusations of mismanagement of the Zoological Society. He concluded the piece, rather, by taking an opportunity to defend himself more generally from “whispered defamations” which had begun to circulate among naturalists about his reputation and character, and which he implicitly suggested Vigors had a close hand in (Swainson 1831b, p. 486). No doubt, the significant attention the public controversy had drawn by that point also provided Swainson with an ideal opportunity for his words to reach a broader audience. Foremost among such rumors were those that called his role as a proto-professional naturalist into question—what Swainson referred to as “opprobrious expressions” made about him “for receiving pecuniary recompense for my writings” (Swainson 1831b, p. 484). The connection of financial gain to scientific publication, some argued, necessarily compromised his capacity for objective research—he would be more interested in ideas that would sell books than in ideas that were true. Swainson countered with a long list of the many respected naturalists who had at one time or another praised him for his “liberality and disinterestedness.” “Eminent” friends and strangers in natural history alike, he insisted, had publicly registered their admiration for his work—appraisals they would not have given if he was truly a “jobber” masquerading as a naturalist. The mark of credibility, for Swainson, was not a complete divestment from any “pecuniary recompense” for one’s scientific publications, but the general esteem of other experts in the field.

Still, such accusations, whether spread by Vigors or not, continued to follow Swainson through the 1830s. In his efforts to support himself, like that for Dionysus Lardner’s widely-circulated *Cabinet Cyclopedia*, Swainson struggled to match a furious pace of deadlines. As a seeming result, Swainson increasingly drew a number of public criticisms for inconsistencies in his nomenclature, most notably from Cambridge geologist Hugh Edwin Strickland. Strickland would soon emerge as one of Swainson and the quinary system’s most vociferous critics, as he began a campaign to standardize a zoological nomenclature in perceived chaos—for which he saw the new natural systems like the quinary as primarily responsible. Strickland’s attacks on Swainson, the leading representative of Macleay’s theory by the mid-1830s, led to both the quinary system’s and Swainson’s ultimate fall into disrepute, which Swainson privately described to William Jackson Hooker as his “voluntary exile” in New Zealand.^44^ The quinary system’s three most active proponents—Macleay, Vigors, and Swainson—had each played central roles in the waning enthusiasm the system faced by 1835, as they became increasingly embroiled in unseemly public controversies. These debates, both against their detractors and amongst themselves, often shocked the gentlemanly audience of British natural history and ultimately diminished each man’s standing in their collective view. The eventual demise of the quinarian system intimately concerns their behavior in those tumultuous years between 1829 and 1831.

## Conclusion: A “Mean Quarrelsome Spirit”

The events of 1829–1831, within and without the field of natural history, are simultaneously the culmination of a process that began in the first decades of the nineteenth century, and a crucial element of any subsequent account of midcentury English science. The eruption of hostilities between zoologists in the early 1830s—and indeed their continuation well through the decade—cannot be attributed to any single primary factor. In certain respects, such controversies constituted the realizations of fears expressed by conservative systematists, who urged their colleagues to tack closely to Linnaean principles of classification in the first two decades of the century, even as those principles came under increasing strain from the veritable “deluge” of novel specimens emanating from the colonies (McOuat 1996, p. 481). This sentiment was perhaps best captured by the relatively obscure pastor and amateur naturalist Edward John Burrow in 1815, who worried that the emerging methods in systematics “have nearly involved the edifice” of a broadly-shared commitment to Linnaean categories and methods “to ruin,” portending an epistemically anarchical and self-perpetuating state of the field in which “scarcely two writers have agreed in their opinions…this general want of concurrence have aggravated the evils that each author has endeavored to remove” (Burrow 1815, pp. v-vi). As adherence to Linnaean classifications waned in England and new theories of how to order nature were developed to replace them, no single system of classification emerged to return a modicum of consensus to the field. This, in turn, was not merely a product of a lack of the conceptual appeal of any one theory—the instability was also reflected in London’s learned societies. The zoologists Linnean Society of London fractured off into the short-lived Zoological Club of the Linnean Society in 1823, frustrated with their parent body’s reticence to entertain new systematic theories. In 1830—the same year that the Zoological Club dissolved, after the majority of its members themselves migrated to Stamford Raffles’s newly-formed Zoological Society of London—Charles Babbage took up his pen against the leadership and management of the Royal Society, which, through a similar reticence to admit debate, had in his estimation unduly inhibited the progress of English science as a whole.

In other respects, the seeds of the hostilities lay not in the content of the ideas themselves, or in the structure of the institutions in which they were aired and discussed, but in the complex network of interpersonal relations that undergirded the relatively small community of London’s elite naturalists—a status defined primarily by membership to the Linnean and Royal Societies of London. The waning momentum of the quinarian system by 1830 cannot be understood without an account of the growing rift between Macleay’s two most prolific proponents, Nicholas Aylward Vigors and William Swainson. Though letters indicate that their initial differences emerged from differing interpretations of their favored theory, such conceptual differences cannot entirely account for the fierce public controversy they would become embroiled in, played out in the pages of the *Magazine of Natural History*, to both the distaste and fascination of its readers. Conversely, declining enthusiasm for the quinary system was not a simple result of eventual recognition of its lack of scientific merit, but rather, was thoroughly intertwined with the manner in which its creator conducted himself in defending it from critics and rivals around the turn of the 1830s, and the manner with which his two most ardent supporters attacked each other shortly afterwards. Those publications, in turn, were simultaneously enabled by a chaotic period that concluded the Georgian period in which calls for both political and scientific reform ignited the passions of the British gentry, and a growing print culture in which editors of periodicals readily published invectives that stood to increase their revenues.^45^

Such were the circumstances that would lead William Swainson to lament the “extraordinary mode of scientific discussions which has of late arisen among us” in 1831, and would lead a young Charles Darwin to complain to his friend John Stevens Henslow in 1836 that “I am out of patience with the Zoologists, not because they are overworked, but for their mean quarrelsome spirit. I went the other evening to the Zoological Soc. where the speakers were snarling at each other, in a manner anything but like that of gentlemen” (Swainson 1831a, p. 97; Darwin 1836). As it turns out, William Sharp Macleay, freshly returned from Havana, was in attendance at that very meeting.^46^ And he was joined, alongside Darwin, by the man that would soon emerge as the quinarian system’s most strident and effective critic to date: Hugh Edwin Strickland. Strickland’s crusade against the new systematic practices of the 1820s and 30s, as Gordon McOuat has powerfully argued, would culminate in the first international rules of nomenclature, a crucial infrastructure of the science to this day (McOuat 1996, pp. 500–515). Macleay—alongside his allies, rivals, and detractors—provided the impetus.

## Footnotes

1 See also Bracy Clark, “Comments on W.S. Macleay’s ‘Remarks on the Identity of certain General Laws.’” SP/206, The Linnean Society of London Archives, London, UK. Henceforth referenced as “Linnean Society.”

2 William Sharp Macleay, “Remarks on Birds.” MS/246, Linnean Society.

3 The arrangement is nearly identical to Linnaeus’s; Macleay and Vigors merely merge his orders *Picae* and *Passares* into *Insessores,* or the “perching birds” (e.g. Vigors 1825, pp. 404–405).

4 See for example William Kirby to William Sharp Macleay, 5 October 1822. ZC/1, Linnean Society.

5 Bentinck was even said to visit Swainson’s humble apartments on one occasion to view his shell collection.

6 Living and working in Liverpool, John Timothy does not seem to have participated very actively in the Society. In a letter to William Swainson, Smith describes his father as a “very old friend,” but was evidently not close enough to the elder Swainson to know that he had a son. See James Edward Smith to William Swainson, 15 January 1816. MS/273, Linnean Society.

7 See for example William Swainson to Alexander Macleay, 10 August 1816. MS/237c, Linnean Society.

8 Smith to Swainson, 15 January 1816, Linnean Society.

9 For a more detailed description of Macleay’s system see Novick (2016).

10 For more on the role and status of keys in classification, see Scharf (2009).

11 While Adrian Desmond has described Bicheno as a reformist moderate, there is also significant evidence of his conservative tendencies (Desmond 1985a, pp. 165–168). On a commission to investigate the condition of the Irish poor in 1836, he dissented from its recommendation to extensively fund public works (Shaw 2004). His letters to the Marquess of Bute in 1840 register his staunch opposition to Chartism. And after he was appointed colonial secretary of Van Diemen’s Land in 1842, colonists nicknamed him “The Old Hen” for his close alignment with the government on issues of parliamentary representation (n.a. 1966). See James Ebenezer Bicheno to the Marquess of Bute, 1840, Bute Estate 2, L83/411, 471. National Library of Wales, Aberystwyth, UK.

12 Minute Book of the Zoological Club of the Linnean Society of London. ZC/3/1, Linnean Society.

13 Ibid.

14 Nicholas Aylward Vigors to William Swainson, “Friday morning” and “Sunday night.” MS/274, Linnean Society.

15 Vigors to Swainson, “Sunday night,” Linnean Society.

16 This essentially comes down to the roles of affinitive and analogous relations in the quinary system. Groups are connected by a single point of affinity, represented by an “osculant group” between them, simultaneously preserving natural continuity and accommodating the existence of discrete groups. For a fuller explanation, see Novick (2016).

17 “At a meeting of the Zoological Club of the Linnean Society of London held at the Rooms of the Society on Tuesday August 26th 1823 at one o’clock.” Minute Book of the Zoological Club of the Linnean Society of London, ZC/3/1, Linnean Society.

18 Though Macleay continued to correspond with London’s naturalists, and wrote regularly for local periodicals, his distance precluded any significant organizational work on behalf of the Club.

19 See also Russell (1917).

20 As discussed below, editors sometimes removed select excerpts from Macleay’s articles in the 1830s, which they found too ungentlemanly for print.

21 The term “*ex Cathedrâ”* was used to describe statements of the Catholic church that were backed by the full authority of the office of the Pope, and thus doctrinally infallible. James Edward Smith, president of the Linnean Society, is almost certainly the authority to which Macleay is referencing.

22 William Sharp Macleay to William Swainson, 27 November 1830. MS/272, Linnean Society; George Johnston to John Edward Gray, 4 December 1829. The British Library Archives, MS/29533, Correspondence of John Edward Gray, The British Library, London, UK. Henceforth referenced as “British Library.”

23 There is an important distinction between the dichotomous system as elaborated by Adrian Hardy Haworth, and John Fleming’s conception of it. By all accounts, Haworth believed his system to be “natural”—to correspond to true categories; Fleming, as evidenced by this essay, did not share this view. For Fleming, the dichotomous system was preferential to the Linnaean, but it was nonetheless “artificial,” in that way that all human classifications necessarily were.

24 For a further elaboration of the dichotomous system, see Bentham (1823). The process, Fleming and Haworth insisted, could be repeated at every hierarchical level of nature—from “animal” and “vegetable” down to “monopetalus” (with a single petal) and “polypetalous” (with many).

25 For Macleay, the editors of the *Quarterly*, too, bore some significant responsibility, whom he accused of failing to notice that “a considerable part of his article is composed of verbatim extracts from ‘The Philosophy of Zoology.’” (p. 432)

26 Duels were considered to be a viable, if extreme, avenue for the settling of interpersonal disputes through the late Georgian period. Just one year before Macleay’s letter, the sitting prime minister, Arthur Wellesley, Duke of Wellington, dueled George Finch-Hatton, Earl of Winchilsea, at Battersea Fields on the south bank of the Thames (n.a. 1829).

27 See William Sharp Macleay to William Swainson, 27 November 1830. MS/272, Linnean Society.

28 William Sharp Macleay to William Swainson, 27 November 1830. MS/272, Linnean Society.

29 William Sharp Macleay to William Swainson, 27 November 1830. MS/272, Linnean Society.

30 Macleay’s private and unpublished manuscripts contain a loose draft of his attack on Fleming, filled with strikethroughs and edits, and drafted explicitly as a letter to Vigors. He elected to frame the article in the genre of a private letter deliberately. See William Sharp Macleay, “On the Latest Device for Bolstering up the Dichotomous System.” MS/243, Linnean Society.

31 The exact volume of sales of particular issues is difficult to determine. *The Philosophical Magazine’s* editors, Richard Taylor and Richard Philips—both naturalists and Fellows of the Linnean Society, may have also held differing opinions on the subject, and settled on the censored publication of Macleay’s continuations as a compromise.

32 William Kirby to William Sharp Macleay, 14 June 1830. MS/237b, Linnean Society.

33 William Kirby to William Sharp Macleay, 14 June 1830. MS/237b, Linnean Society.

34 William Sharp Macleay to Robert Brown, 20 September 1832. MS/32441, Correspondence of Robert Brown, British Library.

35 It should be noted that while Swainson embraced many of the principles of Macleay’s system, he increasingly advanced certain modifications to it in the 1830s. Primary among these were an elevation of the role of analogical relationships and a suggestion that the five members of a quinary group could often be resolved into three. Though Swainson is often considered a “quinarian,” because of his close intellectual kinship with Macleay, it would be more accurate to term his system “circular and trinary.” For this reason, scholars like Novick (2016) argue that Swainson was not in fact a “quinarian” systematist at all.

36 William Swainson to Charles Babbage, 10 January 1832. MS/37166, Letters to Charles Babbage, British Library.

37 William Swainson to Charles Babbage, 8 April 1834. MS/37188, British Library.

38 Charles Babbage to William Swainson, 31 January 1832. MS/270, Linnean Society.

39 Swainson was forced to cease his schooling as a boy due to a speech impediment—see Knight (1985, pp. 83–84).

40 Translated from the French, “Votre livre m’a fait d’autant plus de plaisir que l'exécution m’a paru soignée dans tous les détails,” and “Le public ne peut que gagner à le connaître.”

41 Thomas Stewart Traill to William Swainson, 22 April 1822. MS/274, Linnean Society.

42 “At a meeting of the Zoological Club... on Tuesday August 26th 1823 at one o’clock.” Minute Book of the Zoological Club, ZC/3/1, Linnean Society.

43 Adrian Desmond describes the incident in further detail, citing it as Swainson’s central motivation for joining the growing ranks of Charles Babbage’s “declinists” (Desmond 1985a, pp. 172–174).

44 William Swainson to William Jackson Hooker, 26 June 1839. Ff/135, Director’s Correspondence v. 13, Royal Botanic Gardens, Kew Archives, London, UK.

45 As noted in Lowther (2020) and Secord (2000).

46 “October 25, 1836.” Minutes of Scientific Meetings, The Zoological Society of London Archives, London, UK.
