# Correction: A “Mean Quarrelsome Spirit:” Controversy in British Systematics, 1822–1836

**DOI:** 10.1007/s10739-025-09826-7

**Published:** 2025-05-16

**Authors:** Jordan Thomas Mursinna

**Affiliations:** Department of History, University of California, Berkeley, 3229 Dwinelle Hall, Berkeley, CA 94720-2550 USA

**Correction to: Journal of the History of Biology (2023) 56:673–714** 10.1007/s10739-023-09743-7

In this article, in section ‘The Early Quinarians’, at the beginning of the first full paragraph on page 677, the name ‘William John Swainson’ should have read ‘William Swainson’.

The original article has been corrected.

